# Thermo-Reversible Persistent Phosphorescence Modulation Reveals the Large Contribution Made by Rigidity to the Suppression of Endothermic Intermolecular Triplet Quenching

**DOI:** 10.3389/fchem.2021.788577

**Published:** 2021-11-16

**Authors:** Tomoya Kusama, Shuzo Hirata

**Affiliations:** Department of Engineering Science, University of Electro-Communications, Tokyo, Japan

**Keywords:** persistent room-temperature phosphorescence, triplet quenching, nonradiative deactivation, phase change, diffusion constant, reorganization energy

## Abstract

The suppression of thermally driven triplet deactivation is crucial for efficient persistent room-temperature phosphorescence (*p*RTP). However, the mechanism by which triplet deactivation occurs in metal-free molecular solids at room temperature (RT) remains unclear. Herein, we report a large *p*RTP intensity change in a molecular guest that depended on the reversible amorphous–crystal phase change in the molecular host, and we confirm the large contribution made by the rigidity of the host in suppressing intermolecular triplet quenching in the guest. (*S*)-(−)-2,2′-Bis(diphenylphosphino)-1,1′-binaphthyl ((*S*)-BINAP) was doped as a guest into a highly purified (*S*)-bis(diphenylphosphino)-5,5′,6,6′,7,7′,8,8′-octahydro-1,1′-binaphthyl ((*S*)-H_8_-BINAP) host. It was possible to reversibly form the amorphous and crystalline states of the solid by cooling to RT from various temperatures. The RTP yield (*Φ*_p_) originating from the (*S*)-BINAP was 6.7% in the crystalline state of the (*S*)-H_8_-BINAP host, whereas it decreased to 0.31% in the amorphous state. Arrhenius plots showing the rate of nonradiative deactivation from the lowest triplet excited state (T_1_) of the amorphous and crystalline solids indicated that the large difference in *Φ*_p_ between the crystalline and amorphous states was mostly due to the discrepancy in the magnitude of quenching of intermolecular triplet energy transfer from the (*S*)-BINAP guest to the (*S*)-H_8_-BINAP host. Controlled analyses of the T_1_ energy of the guest and host, and of the reorganization energy of the intermolecular triplet energy transfer from the guest to the host, confirmed that the large difference in intermolecular triplet quenching was due to the discrepancy in the magnitude of the diffusion constant of the (*S*)-H_8_-BINAP host between its amorphous and crystalline states. Quantification of both the T_1_ energy and the diffusion constant of molecules used in solid materials is crucial for a meaningful discussion of the intermolecular triplet deactivation of various metal-free solid materials.

## Introduction

Room-temperature phosphorescence (RTP) with an emission lifetime of more than 100 ms—i.e., persistent RTP (*p*RTP)—from metal-free molecular solids occurs after ceasing exposure to fluorescence-independent excitation light (Clapp. 1939; Zhang et al., 2007; Hirata et al., 2013). Because autofluorescence-independent *p*RTP can be detected using small-scale and low-cost photo detectors, chemicals and materials with *p*RTP characteristics are crucial for state-of-the-art security, sensing, and bioimaging applications (Deng et al., 2013; Zhang et al., 2014; Fateminia et al., 2017; Zhen et al., 2017; Louis et al., 2019; Zhou and Yan, 2019). Efficient *p*RTP produces much brighter persistent emission compared with the general long persistent luminescence from materials that has been reported previously (Bhattacharjee and Hirata, 2020). Therefore, photophysical insight into chemicals and/or materials to access the efficiency and brightness of *p*RTP is crucial. Because *p*RTP from metal-free chromophores is a slow process (Hirata, 2017), the suppression of thermo-driven triplet deactivation is necessary for efficient *p*RTP. However, the mechanism by which the triplet deactivation of metal-free molecular solids occurs at RT remains unclear.

Triplet deactivation includes both intramolecular radiation-less transition from the lowest triplet excited state (T_1_) to the ground state (S_0_) and the T_1_ quenching caused by intermolecular interactions. The rate of the intermolecular radiation-less transition at RT (*k*_nr_(RT)) is related to spin–orbit coupling including vibrations and the energy gap between T_1_ and the S_0_ of the target chromophores (Schlag et al., 1971; Metz et al., 1972; Metz, 1973). However, the rate of the T_1_ quenching caused by intermolecular interactions (*k*_q_(RT)) is based on charge transfer theory (Köhler and Bässler, 2011). In the 1980s, *k*_nr_(RT) and *k*_q_(RT) were considered separately using benzophenone as a guest in polymer matrices, and the *k*_nr_(RT) of benzophenone was found to be almost independent of temperature from 77 K to RT (Horie and Mita, 1982; Horie et al., 1984). Recently, cooperative analysis of optical measurements and quantum chemical calculations have confirmed that the *k*_nr_(RT) values of a variety of heavy atom-free chromophores are less than 10^0^ s^−1^ (Bhattacharjee et al., 2021; Hirata and Bhattacharjee et al., 2021). This indicates that most triplet deactivation of materials is caused by the *k*_q_(RT) (Hirata et al., 2020; Hirata and ; Bhattacharjee et al., 2021). With regard to the *k*_q_(RT) of host–guest molecular solids, it is generally necessary for the host molecules to have a larger T_1_ energy than the metal-based phosphorescence guests to suppress the *k*_q_(RT) (Adachi et al., 2001). However, for heavy atom-free and/or metal-free guests, a long-lived T_1_ at RT generally requires the host molecules to have a considerably greater T_1_ than the guests to significantly suppress the *k*_q_(RT) (Hirata et al., 2013; Totani et al., 2013). Contrary to general discussions about the contribution made by the T_1_ energy difference between the host molecules and the guest chromophores to the *k*_q_(RT), triplet deactivation due to the rigidity of the materials has often been reported, although it is still regarded as phenomenological (Zhao et al., 2020). Although recent analyses suggest that rigidity—including intermolecular interactions—might not be related to *k*_nr_(RT) but to *k*_q_(RT), investigations into the physical factors governing rigidity and the T_1_ energy difference between the host and the guest have not been reported.

Herein, we report the dependency of the thermo-reversible intensity change of green *p*RTP of a guest on the amorphous–crystalline phase change of a host molecule, and determine the contribution made by the rigidity of the host in the suppression of endothermic intermolecular triplet quenching. (*S*)-(−)-2,2′-Bis(diphenylphosphino)-1,1′-binaphthyl ((*S*)-BINAP) was doped into a highly purified (*S*)-bis(diphenylphosphino)-5,5′,6,6′,7,7′,8,8′-octahydro-1,1′-binaphthyl ((*S*)-H_8_-BINAP) host. The host–guest material in the crystalline state produced green *p*RTP under ambient conditions, and the RTP yield (*Φ*_p_(RT)) was 6.7%. However, the *Φ*_p_(RT) of the host–guest material in the amorphous state decreased to 0.31%, even when the materials were stored in a high vacuum. Detailed optical measurements indicated that the large *Φ*_p_(RT) in the crystalline state was caused by both the increase in the triplet generation yield of the (*S*)-BINAP guest and the large decrease of *k*_q_(RT) in the crystalline state compared with in the amorphous state. The comparable T_1_ energy values of the (*S*)-H_8_-BINAP host in both the amorphous and crystalline states suggests that the decrease in *k*_q_(RT) was caused by the suppressed molecular diffusion of the (*S*)-H_8_-BINAP host molecules in the crystalline state. The suppressed molecular diffusion in the crystalline (*S*)-H_8_-BINAP host was observed using molecular dynamic simulation. Thus, suppressed molecular diffusion is a crucial factor for minimizing the endothermic intermolecular triplet energy transfer that induces triplet quenching.

## Results and Discussion

### Thermo-Reversible Intensity Change of Persistent Room-temperature Phosphorescence

**FIGURE 1:**
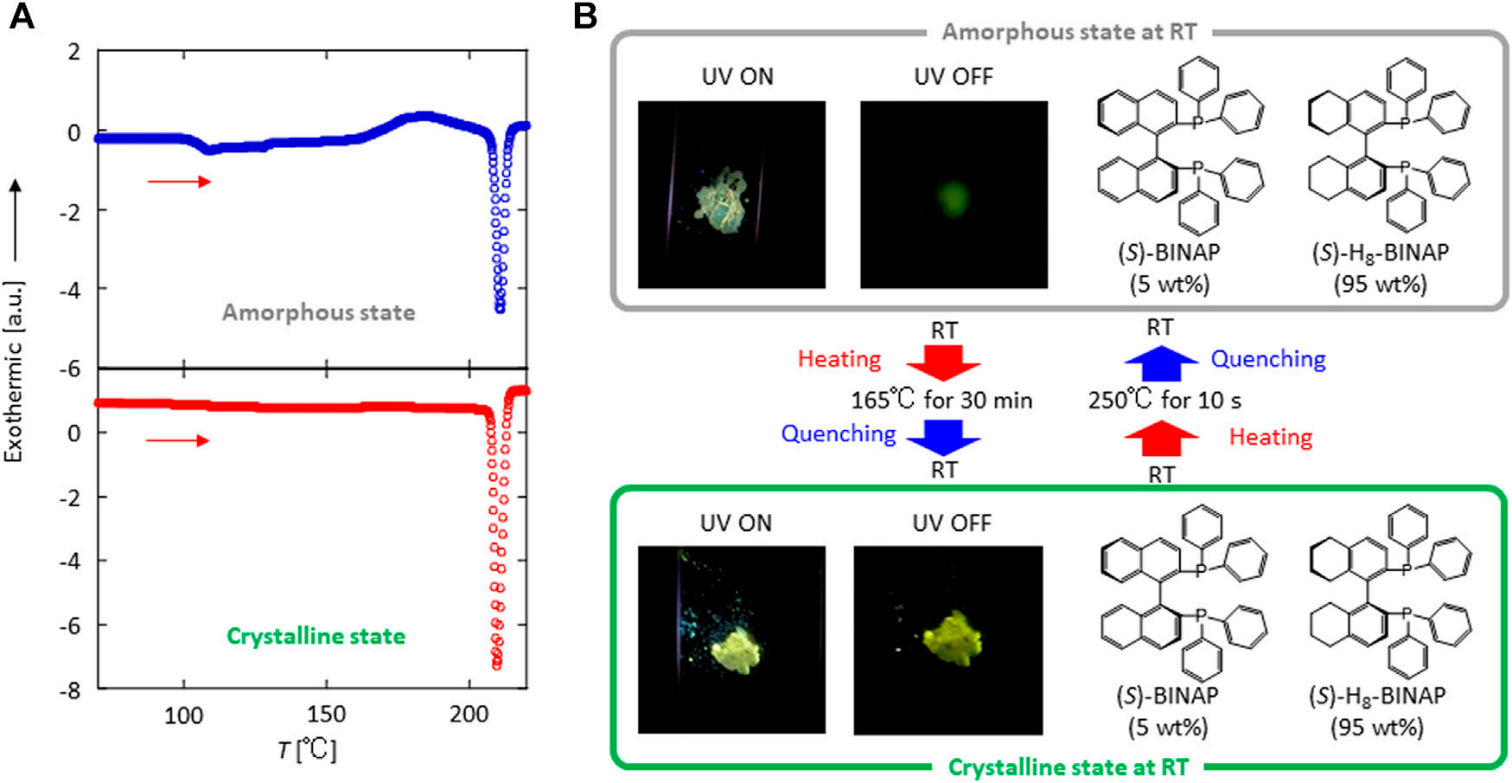
Reversible crystal–amorphous phase change at RT and *p*RTP characteristics of the (*S*)-BINAP doped (*S*)-H_8_-BINAP solid. **(A)** Differential scanning calorimetry characteristics of amorphous state **(upper)** and crystalline state **(lower)** when heated at 5°C/min. **(B)** Emission behavior during and immediately after excitation by exposure to radiation (330 nm) of amorphous **(upper)** and crystalline **(lower)** solids. ((*S*)-BINAP = (*S*)-(−)-2,2′-bis(diphenylphosphino)-1,1′-binaphthyl (*S*)-H_8_-BINAP = (*S*)-bis(diphenylphosphino)-5,5′,6,6′,7,7′,8,8′-octahydro-1,1′-binaphthyl; *p*RTP = persistent room-temperature phosphorescence).

Commercially available (*S*)-H_8_-BINAP crystals often generate green *p*RTP after ultraviolet (UV) excitation has ceased (Supplementary Figure S1A) because of the presence of (*S*)-BINAP as an impurity (Supplementary Figure S2). After careful repeated purification of the (*S*)-H_8_-BINAP using silica column chromatography, however, we noted that the purified crystals of (*S*)-H_8_-BINAP did not produce *p*RTP (Supplementary Figure S1B) (Hirata et al., 2020). First (*S*)-BINAP powder was dissolved in molten pure (*S*)-H_8_-BINAP at 220°C. Next, the molten materials were placed on a quartz substrate on a hotplate at 250°C which is higher temperature than a melting point of 208°C of (*S*)-H_8_-BINAP host (Supplementary Figure S3), and the substrate was quenched to room temperature (RT) to prepare an amorphous 5 wt% (*S*)-BINAP-doped (*S*)-H_8_-BINAP film. The resulting amorphous film of 5 wt% (*S*)-BINAP-doped (*S*)-H_8_-BINAP produced markedly weak *p*RTP after ceasing UV excitation (Figure 1B; top). The weak *p*RTP of the amorphous 5 wt% (*S*)-BINAP-doped (*S*)-H_8_-BINAP film did not increase under vacuum. The amorphous form of 5 wt% (*S*)-BINAP-doped (*S*)-H_8_-BINAP has a glass transition temperature of 104°C and crystallizes at 165°C (Figure 1A; top). After crystallization at 165°C for 30 min, the produced 5 wt% (*S*)-BINAP-doped (*S*)-H_8_-BINAP crystalline films (Figure 1A; lower) produced much brighter green *p*RTP compared with the amorphous form after exposure to excitation light of 330 nm had ceased (Figure 1B; lower).

**FIGURE 2:**
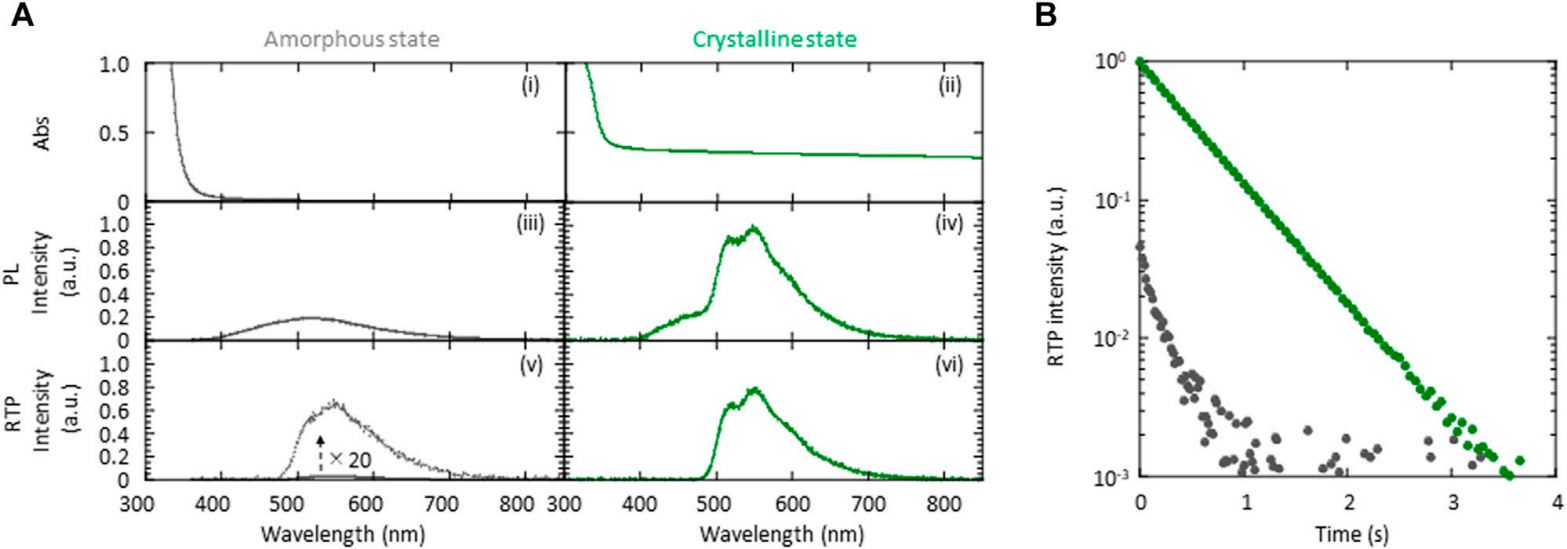
Absorption and emission characteristics of 5 wt% (*S*)-BINAP-doped (*S*)-H_8_-BINAP films. **(A)** Absorption (i, ii), steady-state RT emission (iii, iv), and RTP (v, vi) spectra of amorphous (gray) and crystalline (green) films. **(B)** RTP decay characteristics of amorphous (gray) and crystalline (green) films. The RTP intensity immediately after ceasing excitation in the crystalline state was normalized to one and the initial intensity of RTP immediately after ceasing excitation in the amorphous state was set by considering the difference in *Φ*_p_(RT) between the crystalline and amorphous states. In **(A)** and **(B)**, the excitation wavelength used for emission measurements was 330 nm. (RT = room-temperature; RTP = room-temperature phosphorescence; (*S*)-BINAP = (*S*)-(−)-2,2′-bis(diphenylphosphino)-1,1′-binaphthyl (*S*)-H_8_-BINAP = (*S*)-bis(diphenylphosphino)-5,5′,6,6′,7,7′,8,8′-octahydro-1,1′-binaphthyl).

More detailed optical measurements were performed to investigate the differences in the *p*RTP characteristics between the crystalline and amorphous states of 5 wt% (*S*)-BINAP-doped (*S*)-H_8_-BINAP solid. Both the crystalline and amorphous 5 wt% (*S*)-BINAP-doped (*S*)-H_8_-BINAP films absorbed radiation at 330 nm ((i) and (ii) in Figure 2A). The amorphous 5 wt% (*S*)-BINAP-doped (*S*)-H_8_-BINAP film produced a fluorescence spectrum beginning at approximately 400 nm ((iii) in Figure 2A), and the steady-state RT emission yield was 2.5%. Immediately after excitation by exposure to UV radiation ceased, a weak green *p*RTP remained ((v) in Figure 2A). The quantum yield of phosphorescence at RT (*Φ*_p_(RT)) was estimated to be 0.31% by comparing the intensity of the steady-state RT emission spectrum ((iii) in Figure 2A) with the RTP spectral intensity immediately after ceasing excitation ((v) in Figure 2A). The RTP decay had multi-exponential characteristics (gray in Figure 2B). For the crystalline 5 wt% (*S*)-BINAP-doped (*S*)-H_8_-BINAP films, the larger increase of absorbance at wavelengths less than 350 nm resulted from the absorption of (*S*)-BINAP and (*S*)-H_8_-BINAP ((ii) in Figure 2A). An increase of the baseline at wavelengths longer than 400 nm was caused by light scattering arising from the polycrystalline films. In contrast to the amorphous state, the crystalline 5 wt% (*S*)-BINAP-doped (*S*)-H_8_-BINAP film had two large peaks at 510 and 545 nm under steady-state UV excitation at 330 nm in addition to fluorescence spectra commencing at approximately 400 nm ((iv) in Figure 2A). The steady-state RT emission yield was 9.8%. After ceasing excitation, a large RTP spectra with peaks at 510 and 545 nm remained ((vi) in Figure 2A). *Φ*_p_(RT) was determined to be 6.7% by comparing the spectral intensity of steady-state RT emission ((iv) in Figure 2A) with the RTP spectral intensity immediately after ceasing excitation ((vi) in Figure 2A). The RTP had single exponential decay characteristics (green in Figure 2B). Thus, the intensity of the *p*RTP produced by the 5 wt% (*S*)-BINAP-doped (*S*)-H_8_-BINAP solid differed by approximately 20 times when comparing the crystal and amorphous states of the materials. The large *Φ*_p_(RT) of (*S*)-BINAP-doped (*S*)-H_8_-BINAP crystalline films does not change at concentrations of (*S*)-BINAP from 5 wt% to 15 wt%, but substantially decreased at higher concentrations of (*S*)-BINAP (Supplementary Figure S4 and Supplementary Table S1).

## Fundamental Photophysical Properties

**FIGURE 3:**
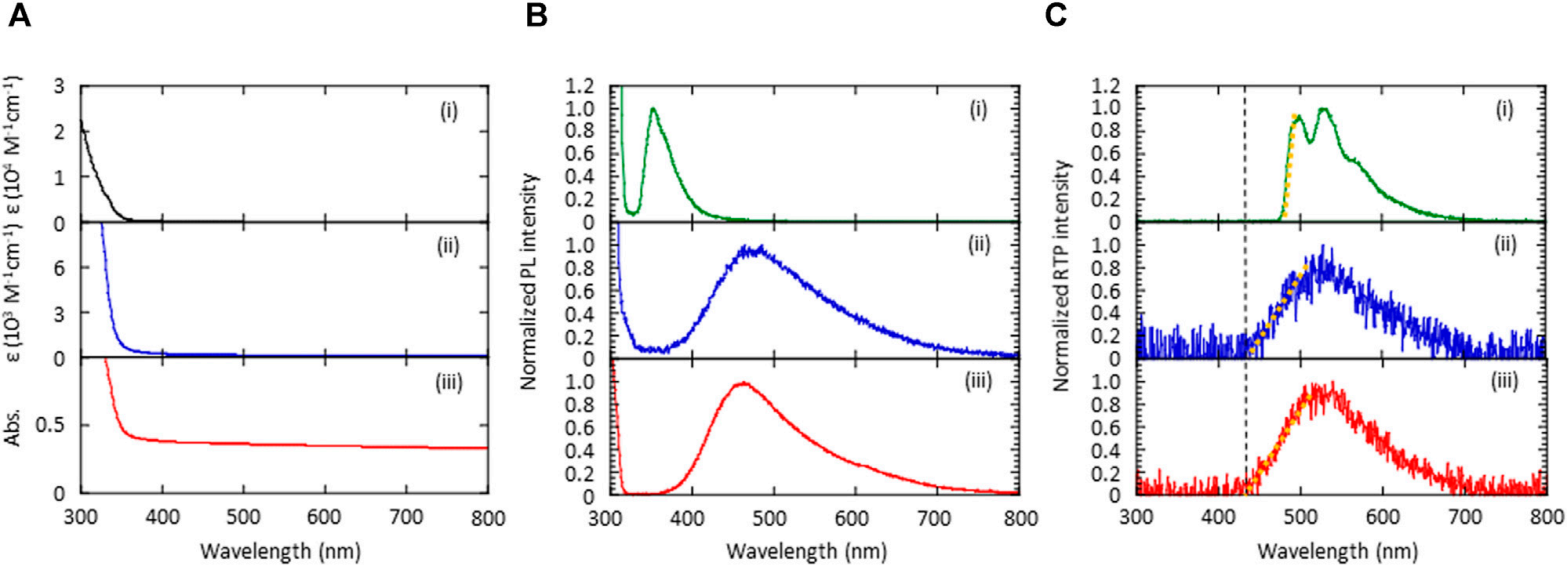
Absorption and emission spectra of (*S*)-BINAP molecularly dispersed in 2Me-THF (i), an amorphous (*S*)-H_8_-BINAP film (ii), and a crystalline (*S*)-H_8_-BINAP film (iii). **(A)** Absorption spectra. **(B)** Fluorescence spectra obtained by excitation at 300 nm at RT. **(C)** Phosphorescence spectra obtained after ceasing excitation at 300 nm at 77 K. The orange dotted lines in the graph were used to determine the onset energy of T_1_. (2Me-THF = 2-methyltetrahydrofuran; RT = room-temperature; (*S*)-BINAP = (*S*)-(−)-2,2′-bis(diphenylphosphino)-1,1′-binaphthyl (*S*)-H_8_-BINAP = (*S*)-bis(diphenylphosphino)-5,5′,6,6′,7,7′,8,8′-octahydro-1,1′-binaphthyl).

The absorption and emission characteristics of the (*S*)-BINAP guest and the (*S*)-H_8_-BINAP host were measured to determine the mechanism by which green *p*RTP was generated by the 5 wt% (*S*)-BINAP-doped (*S*)-H_8_-BINAP films. When dissolved in 2-methyltetrahydrofuran (2Me-THF), the (*S*)-BINAP guest produced an absorption spectrum at wavelengths of less than 350 nm, and the molar absorption coefficient (*ε*) at 330 nm was 6.1 × 10^3^ M^−1^ cm^−1^ (Figure 3A; (i)). The neat amorphous film of (*S*)-H_8_-BINAP had a long small tail of absorption in the 350–400 nm range, and an ε at 330 nm of 6.4 × 10^2^ M^−1^ cm^−1^ when the density of the amorphous (*S*)-H_8_-BINAP solid was considered to be approximately 1 (Figure 3A; (ii)). The comparison of ε between the (*S*)-BINAP guest and the (*S*)-H_8_-BINAP host indicated that the (*S*)-BINAP guest absorbed 33% of the excitation light at 330 nm when the amorphous 5 wt% (*S*)-BINAP-doped (*S*)-H_8_-BINAP films were irradiated with excitation light. In the neat crystalline film of (*S*)-H_8_-BINAP, light scattering increased from the baseline at wavelengths longer than 350 nm, whereas (*S*)-H_8_-BINAP exhibited marked absorption at wavelengths less than 350 nm (Figure 3A; (iii)).

The (*S*)-BINAP guest dissolved in 2Me-THF produced a fluorescence spectrum with a peak at 353 nm (Figure 3B; (i)). The amorphous and crystalline (*S*)-H_8_-BINAP hosts produced broad fluorescence spectra with onset energies at approximately 400 nm (Figure 3B; 2) and (iii)). Thus, the energy of the lowest singlet excited state (S_1_) of the amorphous and crystalline (*S*)-H_8_-BINAP hosts was slightly smaller than that of the molecularly dispersed (*S*)-BINAP. However, the T_1_ energy of the molecularly dispersed (*S*)-BINAP was much smaller than that of the amorphous and crystalline (*S*)-H_8_-BINAP hosts. The (*S*)-BINAP molecularly dispersed in 2Me-THF produced green persistent phosphorescence at 77 K immediately after ceasing excitation at 330 nm (Figure 3C; (i)). The spectral shape produced by the phosphorescence and the energy of the molecularly dispersed (*S*)-BINAP was comparable to those of the amorphous and crystalline 5 wt% (*S*)-BINAP-doped (*S*)-H_8_-BINAP films. Because neat solid (*S*)-BINAP does not produce RTP at all, even when it is in a vacuum, the green *p*RTP of the amorphous and crystalline 5 wt% (*S*)-BINAP-doped (*S*)-H_8_-BINAP films was caused by the molecularly dispersed (*S*)-BINAP. Although high-concentration doping is not generally appropriate when different chromophores are doped into different crystalline molecules, the similar sizes and structures of (*S*)-BINAP and (*S*)-H_8_-BINAP may allow the efficient replacement of (*S*)-H_8_-BINAP by (*S*)-BINAP in a (*S*)-H_8_-BINAP crystalline lattice. At 77K, both the amorphous and crystalline neat films of (*S*)-H_8_-BINAP produced broad phosphorescence spectra with onset energies of approximately 425 nm (Figure 3C; 2) and (iii)). The comparable S_1_ and T_1_ energies between the amorphous and crystalline (*S*)-H_8_-BINAP hosts do not satisfactorily explain the large difference in the *Φ*_p_(RT) between the (*S*)-BINAP in the amorphous (*S*)-H_8_-BINAP host and that in the crystalline (*S*)-H_8_-BINAP host. Appropriate discussions about *Φ*_p_(RT) generally require investigation of both the generation yield of T_1_ and the T_1_-S_0_ processes (Hirata, 2017). Therefore, the following two sections comprise a discussion of these two points.

## Triplet Generation Scheme

**FIGURE 4:**
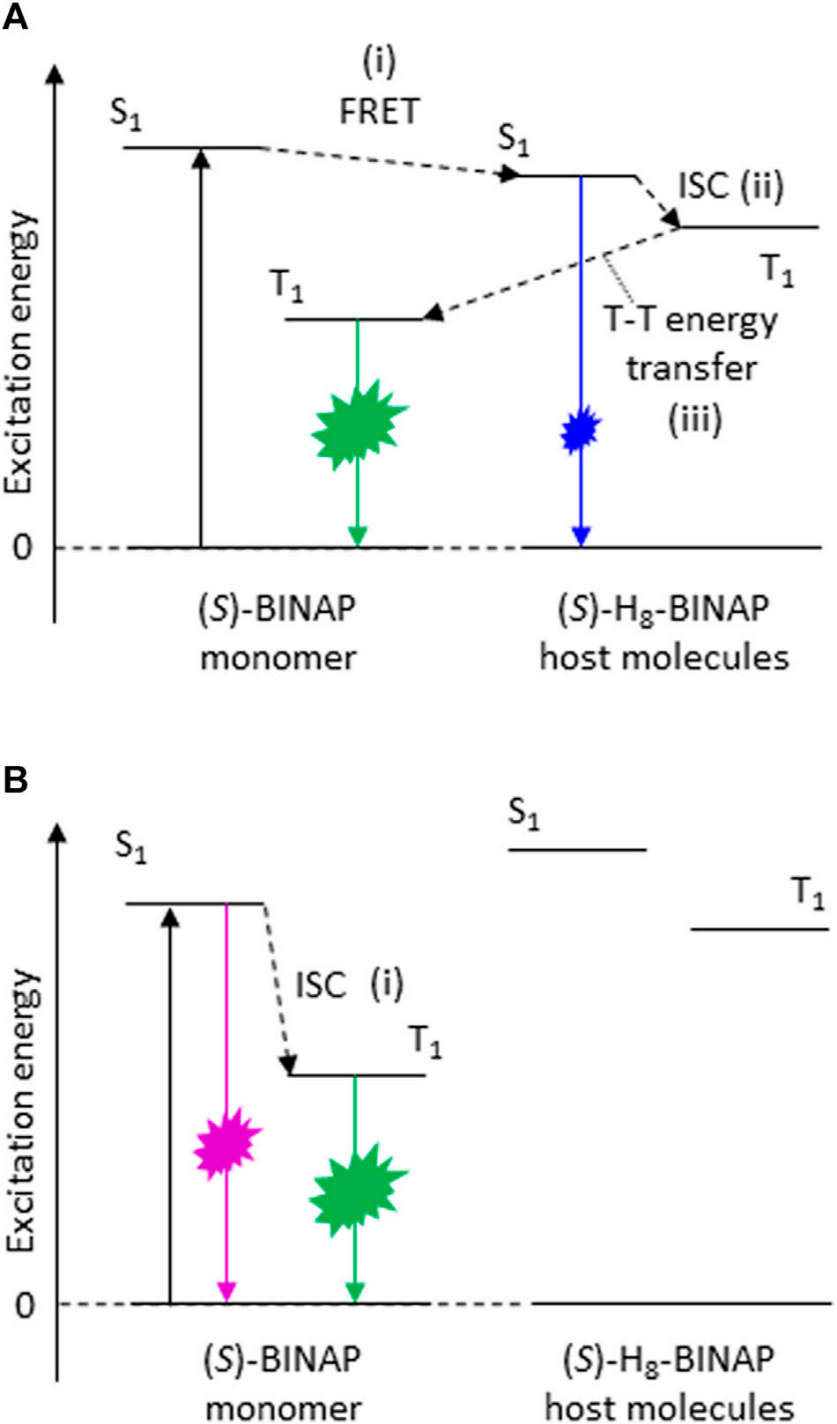
Scheme showing T_1_ generation in (*S*)-BINAP. **(A)** In (*S*)-H_8_-BINAP hosts. **(B)** In amorphous *β*-estradiol host. ((*S*)-BINAP = (*S*)-(−)-2,2′-bis(diphenylphosphino)-1,1′-binaphthyl (*S*)-H_8_-BINAP = (*S*)-bis(diphenylphosphino)-5,5′,6,6′,7,7′,8,8′-octahydro-1,1′-binaphthyl).

The T_1_ generation mechanism and the yield of the (*S*)-BINAP doped into the (*S*)-H_8_-BINAP host were investigated. Again, we note that the (*S*)-BINAP dissolved in 2Me-THF produced fluorescence with a peak wavelength at 353 nm (Figure 3B, (i)). However, the peak disappeared, and a broad-fluorescence spectra with an onset energy at approximately 400 nm appeared when the (*S*)-BINAP was doped into the amorphous and crystalline (*S*)-H_8_-BINAP hosts ((iii) and (iv) of Figure 2A, respectively). Those fluorescence spectra are comparable to the fluorescence spectra of the amorphous and crystalline (*S*)-H_8_-BINAP hosts ((ii) and (iii) of Figure 3B, respectively). The S_1_ energy of (*S*)-BINAP was transferred to the amorphous and crystalline (*S*)-H_8_-BINAP hosts via fluorescence resonance energy transfer (FRET) ((i) in Figure 4A). The generated S_1_ energy of the (*S*)-H_8_-BINAP hosts allowed intersystem crossing (ISC) from S_1_ to generate T_1_ in the (*S*)-H_8_-BINAP hosts ((ii) in Figure 4A). Finally, the generated T_1_ in the (*S*)-H_8_-BINAP hosts was effectively trapped by the T_1_ of the (*S*)-BINAP, because the T_1_ of the (*S*)-BINAP was much less than that of the (*S*)-H_8_-BINAP hosts ((iii) in Figure 4A).

The T_1_ generation yield of the (*S*)-BINAP in the (*S*)-H_8_-BINAP host (*Φ*_t_) via the processes from (i) to (iii) in Figure 4A has the following relationship with *Φ*_p_(77 K):

$$\Phi_{p}\left(\mathrm{77}K\right)\;=\;\Phi_{t}k_{p}\tau_{p}\left(\mathrm{77}K\right)$$ (1)

where *k*_p_ is the rate constant of phosphorescence of the (*S*)-BINAP guest. (*S*)-BINAP doped into an amorphous *β*-estradiol host produced a fluorescence spectrum at 355 nm (blue in Supplementary Figure S2A), and generated green *p*RTP (green in Supplementary Figure S5A,B). In addition, the fluorescence energy of the (*S*)-BINAP doped into the amorphous *β*-estradiol was comparable to that of the (*S*)-BINAP dissolved in 2Me-THF. Therefore, the T_1_ of the (*S*)-BINAP doped into the amorphous *β*-estradiol was formed via the ISC from S_1_ to the triplet states ((i) in Figure 4B). The *Φ*_p_(RT) of the (*S*)-BINAP doped into the amorphous *β*-estradiol can be expressed as (Hirata, 2017):

$$\Phi_{p}\left(\mathrm{RT}\right)\;=\;\Phi_{\mathrm{isc}}k_{p}\tau_{p}\left(\mathrm{RT}\right)$$ (2)

where *Φ*_isc_ is the ISC yield from S_1_ to the triplet states. The *Φ*_isc_ of the molecularly dispersed (*S*)-BINAP was quantified as 0.29 using a transient absorption technique (Supplementary Figure S6; Supplementary Table S2) (Bhattacharjee and Hirata, 2020). Because 0.3 wt% (*S*)-BINAP doped into *β*-estradiol had an *Φ*_p_(RT) of 0.052 and a *τ*_p_(RT) of 0.72 s (Supplementary Figure S5C), the *k*_p_ of (*S*)-BINAP was quantified as 0.25 s^−1^ according to the values of *Φ*_isc_, *Φ*_p_(RT), and *τ*_p_(RT) in Eq. (2). The optically determined *k*_p_ was comparable to 0.22 s^−1^, which was the *k*_p_ value calculated using the optimized T_1_ geometry of (*S*)-BINAP. In addition, the locally excited transition characteristics between T_1_ and S_0_, which can be used to explain the optically observed vibrational shape of the *p*RTP spectra of (*S*)-BINAP, were calculated for the optimized T_1_ geometry of (*S*)-BINAP (Supplementary Figure S7A). The calculated T_1_-S_0_ transition energy based on the optimized T_1_ geometry of (*S*)-BINAP provides a simple statistical explanation for the green color of the *p*RTP of the (*S*)-BINAP (Supplementary Figure S7B). The *Φ*_p_(77K) and *τ*_p_(77K) values of the amorphous 5 wt% (*S*)-BINAP-doped (*S*)-H_8_-BINAP film were 0.023 and 0.63 s, respectively. The optically determined *k*_p_, the *Φ*_p_(77K), and the *τ*_p_(77K) were substituted into Eq. 1 to determine the *Φ*_t_ = 0.15 of the amorphous 5 wt% (*S*)-BINAP-doped (*S*)-H_8_-BINAP film (Table 1). Similarly, the *Φ*_p_(77K) and *τ*_p_(77K) values of the crystalline 5 wt% (*S*)-BINAP-doped (*S*)-H_8_-BINAP film were 0.125 and 0.75 s, respectively. The *k*_p_, the *Φ*_p_(RT), and the *τ*_p_(RT) were substituted into Eq. 1 to determine the *Φ*_t_ = 0.68 of the crystalline 5 wt% (*S*)-BINAP-doped (*S*)-H_8_-BINAP film (Table 1). Hence the approximate 4.5 times difference in *Φ*_t_ between the crystalline and amorphous 5 wt% (*S*)-BINAP-doped (*S*)-H_8_-BINAP films. However, the difference in *Φ*_t_ was still small compared with the approximate 20 times difference in *Φ*_p_(RT) between the crystalline and amorphous 5 wt% (*S*)-BINAP-doped (*S*)-H_8_-BINAP films. Therefore, further investigation about the mechanism of transition from T_1_ to S_0_ in the films is required.

**TABLE 1:** Photophysical values relating to phosphorescence of 5 wt% (*S*)-BINAP-doped (*S*)-H_8_-BINAP solids in crystalline and amorphous states.

Phase of host	*Φ*_t_ ^a^	*Φ*_p_(RT)	*Φ*_p_(77 K)	*τ*_p_(77 K)	*k*_nr_(RT) + *k*_q_(RT)^b^	*k*_nr_(RT)^c^	*k*_q_(RT)^d^
	(%)	(%)	(%)	(s)	(s^−1^)	(s^−1^)	(s^−1^)
Crystal	68	6.7	12.5	0.75	2.24	1.65	0.59
Amorphous	15	0.31	2.3	0.63	11.5	2.15	9.36

a Values determined using Eq. 1.

b Values calculated by substituting Φ_t_, Φ_p_(RT), and τ_p_(77 K) into Eq. 4.

c Values determined using fitting lines in Figure 5B.

d Values determined by subtracting *k*_nr_(RT) determined using fitting lines in Figure 5B from k_nr_(RT) + k_q_(RT).

(*S*)-BINAP = (*S*)-(−)-2,2′-bis(diphenylphosphino)-1,1′-binaphthyl (*S*)-H_8_-BINAP = (*S*)-bis(diphenylphosphino)-5,5′,6,6′,7,7′,8,8′-octahydro-1,1′-binaphthyl; RT = room temperature.

## Large Different Triplet Deactivation Depending on the Phase Change of the Host

To investigate the large difference in *Φ*_p_(RT) between the amorphous and crystalline states of the 5 wt% (*S*)-BINAP-doped (*S*)-H_8_-BINAP, the temperature dependence of the emission characteristics of the two materials was determined. The fluorescence characteristics were almost independent of temperature in both the amorphous and crystalline states (Supplementary Figure S8), whereas there was a large difference between the temperature dependence of the phosphorescence characteristics of the amorphous and crystalline states (Figure 5A and Supplementary Figure S9) of the 5 wt% (*S*)-BINAP-doped (*S*)-H_8_-BINAP solid. The *Φ*_p_ of the crystalline 5 wt% (*S*)-BINAP-doped (*S*)-H_8_-BINAP decreased from 12.5 to 6.7% as the temperature increased from 77 K to RT (red in Figure 5A). In contrast, the *Φ*_p_ of the amorphous 5 wt% (*S*)-BINAP-doped (*S*)-H_8_-BINAP decreased markedly from 2.3 to 0.31% as the temperature increased from 77 K to RT (blue in Figure 5A). The phosphorescence lifetime at *T* (*τ*_p_(*T*)) is generally expressed as (Hirata, 2017):

$$\tau_{p}\left(T\right)\;=1/\left[k_{p}+k_{\mathrm{nr}}\left(T\right)\;+k_{q}\left(T\right)\right]\;$$ (3)

**FIGURE 5:**
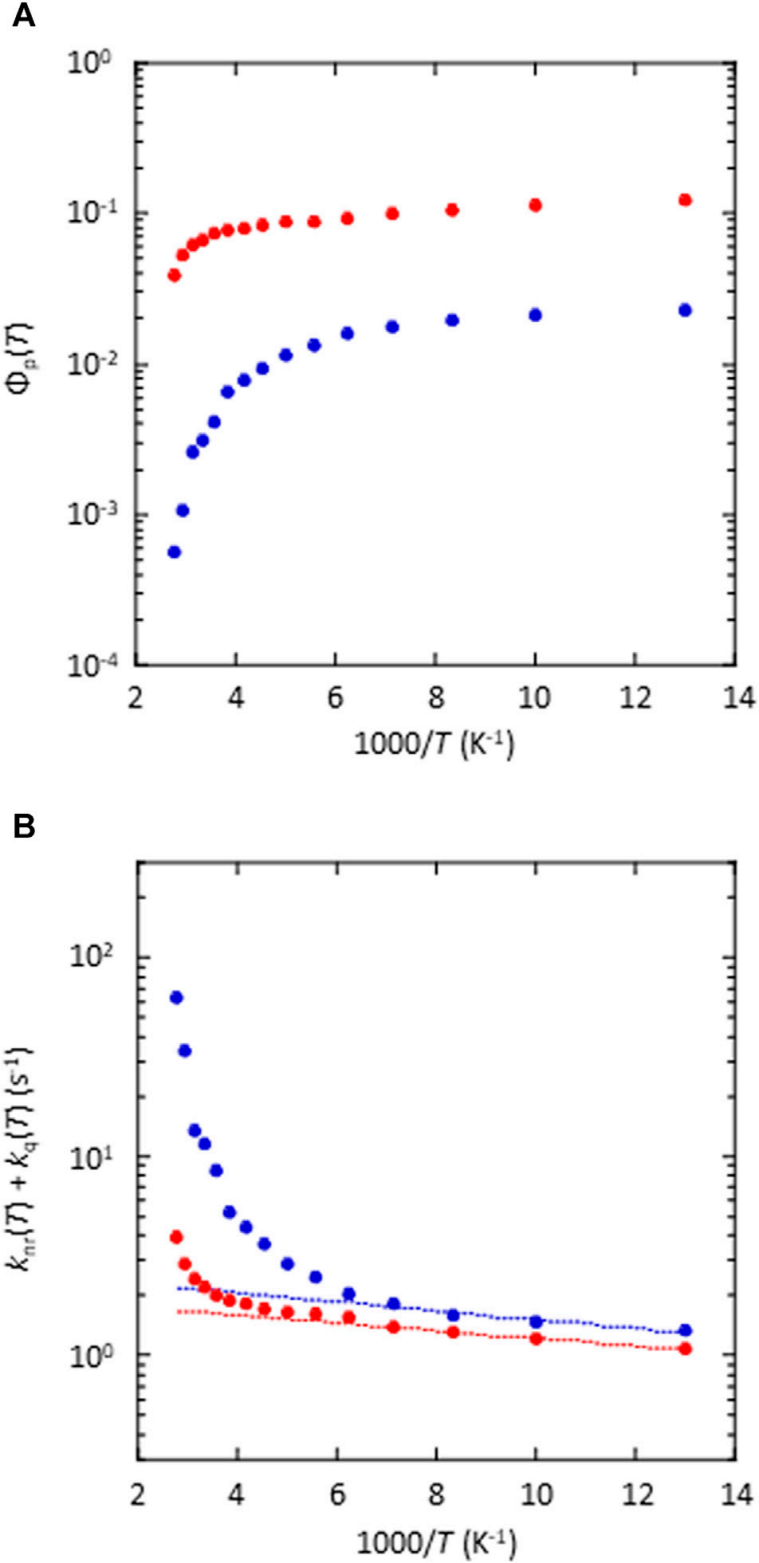
Temperature dependence of phosphorescence and nonradiative deactivation of amorphous (blue) and crystalline (red) 5 wt% (*S*)-BINAP-doped (*S*)-H_8_-BINAP. **(A)** Temperature dependence of *Φ*_p_(*T*). The excitation wavelength was 330 nm. **(B)** Temperature dependence of *k*_nr_(T) + *k*_q_(T). The dashed lines are fitting lines for *k*_nr_(*T*), which was determined according to the exponential function for data from 77K to 140 K. ((*S*)-BINAP = (*S*)-(−)-2,2′-bis(diphenylphosphino)-1,1′-binaphthyl (*S*)-H_8_-BINAP = (*S*)-bis(diphenylphosphino)-5,5′,6,6′,7,7′,8,8′-octahydro-1,1′-binaphthyl).

The following equation can be produced from Eq. 1 and Eq. 3:

$$k_{\mathrm{nr}}\left(T\right)\;+k_{q}\left(T\right)\;=\;\Phi_{t}k_{p}/\Phi_{p}\left(T\right)\;-k_{p}$$ (4)

For the amorphous 5 wt% (*S*)-BINAP-doped (*S*)-H_8_-BINAP film, *Φ*_t_ = 0.15, *k*_p_ = 0.25 s^−1^, and *Φ*_p_(*T*) (blue plots in Figure 5A) were substituted into Eq. 4 to determine the *k*_nr_(*T*) + *k*_q_(*T*) of the molecularly dispersed (*S*)-BINAP in the amorphous (*S*)-H_8_-BINAP host (blue plots in Figure 5B). For the 5 wt% (*S*)-BINAP doped into the crystalline (*S*)-H_8_-BINAP film, *Φ*_t_ = 0.68, *k*_p_ = 0.25 s^−1^, and *Φ*_p_(*T*) (red plots in Figure 5A) were substituted into Eq. 4 to determine the *k*_nr_(*T*) + *k*_q_(*T*) of the molecularly dispersed (*S*)-BINAP in the crystalline (*S*)-H_8_-BINAP host (red plots in Figure 5B).

The *k*_nr_(*T*) + *k*_q_(*T*) of (*S*)-BINAP did not increase significantly when (*S*)-BINAP was doped into the crystalline (*S*)-H_8_-BINAP host (red in Figure 5B). However, the *k*_nr_(*T*) + *k*_q_(*T*) of (*S*)-BINAP increased markedly in the amorphous (*S*)-H_8_-BINAP host (blue in Figure 5B). We note that *k*_nr_(*T*) + *k*_q_(*T*) was comparable in the low-temperature range, whereas it was markedly different in the high-temperature range. In previous research, the very small increase in *k*_nr_(*T*) + *k*_q_(*T*) in the low-temperature range was often caused by *k*_nr_(*T*) (Hirata et al., 2013; Kwon et al., 2015). The lack of a marked increase in *k*_nr_(*T*) as the temperature increases has been confirmed from experimental and theoretical viewpoints using a variety of heavy atom-free chemical backbones (Bhattacharjee et al., 2021; Hirata and Bhattacharjee, 2021). Therefore, the lack of a marked increase in *k*_nr_(*T*) in (*S*)-BINAP is plausible. Indeed, the theoretical calculations that depend on spin–orbit coupling with consideration for vibrations support the idea that the *k*_nr_(*T*) in (*S*)-BINAP hardly increased from 77 K to RT (Supplementary Figure S10). Therefore, *k*_nr_(*T*) is almost independent of intermolecular interactions between (*S*)-BINAP and (*S*)-H_8_-BINAP. Because the comparable *k*_nr_(*T*) values of (*S*)-BINAP in different phase conditions were estimated (Figure 5B), the large difference in *k*_nr_(*T*) + *k*_q_(*T*) at high temperatures was caused by intermolecular processes, i.e., *k*_q_(*T*).

## Discussion of the Driving Force Behind Intermolecular Triplet Quenching

Triplet quenching caused by endothermic triplet–triplet energy transfer has been reported for metal-free and/or heavy atom-free chromophores with small *k*_p_ and *k*_nr_(RT) values, even when the T_1_ energy of the host is much larger than that of the guest, and the solid materials are under high vacuum (Hirata et al., 2013; Totani et al., 2013). In research on organic light emitting diodes, *k*_q_(RT) values of varying magnitudes are often discussed in terms of the difference in the T_1_ energy between the guest and host (Δ*E*) (Adachi et al., 2001). However, the absence of a significant difference between the onset energy values of the phosphorescence spectra of the amorphous and crystalline states of (*S*)-H_8_-BINAP (Figure 3C; (ii) and (iii)) indicates that the T_1_ energy of the neat (*S*)-H_8_-BINAP solid hardly changed between those states. Because the onset energy of phosphorescence of the (*S*)-BINAP guest also hardly changed between the amorphous and crystalline states of (*S*)-H_8_-BINAP host, the Δ*E* of the 5 wt% (*S*)-BINAP-doped (*S*)-H_8_-BINAP was comparable between those states. Therefore, other physical viewpoints are crucial for explaining the difference in *k*_q_(RT) values between the amorphous and crystalline states of 5 wt% (*S*)-BINAP-doped (*S*)-H_8_-BINAP solid.

It may be necessary to consider other diffusion-limited processes to determine potential factors that contribute to *k*_q_(RT). Although endothermic processes are not generally rapid, the diffusion process in a solid state is potentially slower than a slow endothermic process. Because this condition satisfies the definition of a diffusion-limited process, the observable rate constant *k*_q_(RT) can be expressed using the formula for a general diffusion-limited process (Fukuzumi et al., 2003; Chakraborty et al., 2006):

$$k_{q}\left(T\right)\;=\;\frac{k_{\mathrm{diff}}\left(T\right)k_{\mathrm{et}}\left(T\right)}{\left[k_{\mathrm{diff}}\left(T\right)\;+\;k_{\mathrm{et}}\left(T\right)\right]}$$ (5)

where *k*_diff_(*T*) is the diffusion rate constant at *T* and *k*_et_(*T*) is the rate constant of triplet deactivation via triplet–triplet energy transfer from guest to host. *k*_diff_(*T*) is generally expressed as: (Horie et al., 1984)

$$k_{\mathrm{diff}}\left(T\right)\;=\;4\pi \mathrm{RD}\left(T\right)N[C]$$ (6)

where *R* is the reaction radius between guest and host, *D*(*T*) is the diffusion coefficient at *T* K, *N* is the Avogadro number, and [*C*] is the concentration of host. Because Dexter energy transfer is often discussed using the double electron transfer model according to Marcus-based theory (Dexter, 1953; Marcus, 1956; Closs et al., 1989; Köhler and Bässler, 2011), *k*_et_(*T*) may be expressed based on the following Marcus formula:

$$k_{\mathrm{et}}\left(T\right)\;=\nu \left(\frac{1}{\sqrt{4\mathrm{k\lambda T}}}\right)\exp\left[-\frac{{\left(\lambda +\;\mathrm{\Delta E}\right)}^{2}}{4\mathrm{\lambda kT}}\right]$$ (7)

where *ν* is the frequency of the motion in the reactant potential well between guest and host, *λ* is the reorganization energy for triplet–triplet energy transfer, and *k* is the Boltzman constant. Eq. 5 and Eq. 6 indicate that *D*(RT) is almost proportional to *k*_q_(RT) when *k*_diff_(RT) is much less than *k*_et_(RT). Although the relationship between *k*_q_(RT) and Δ*E* is mostly considered with regard to solid molecular materials in the field of organic light emitting diodes (Adachi et al., 2001; Hirata et al., 2013; Notsuka et al., 2017), Eqs. 5–7 indicate that an increase in λ and a decrease in *D*(RT) also contribute to the suppression of *k*_q_(RT). Therefore, consideration of Δ*E*, *λ*, and *D*(RT) is necessary for a discussion of the difference in *k*_q_(RT) values between amorphous and crystalline states.

**FIGURE 6:**
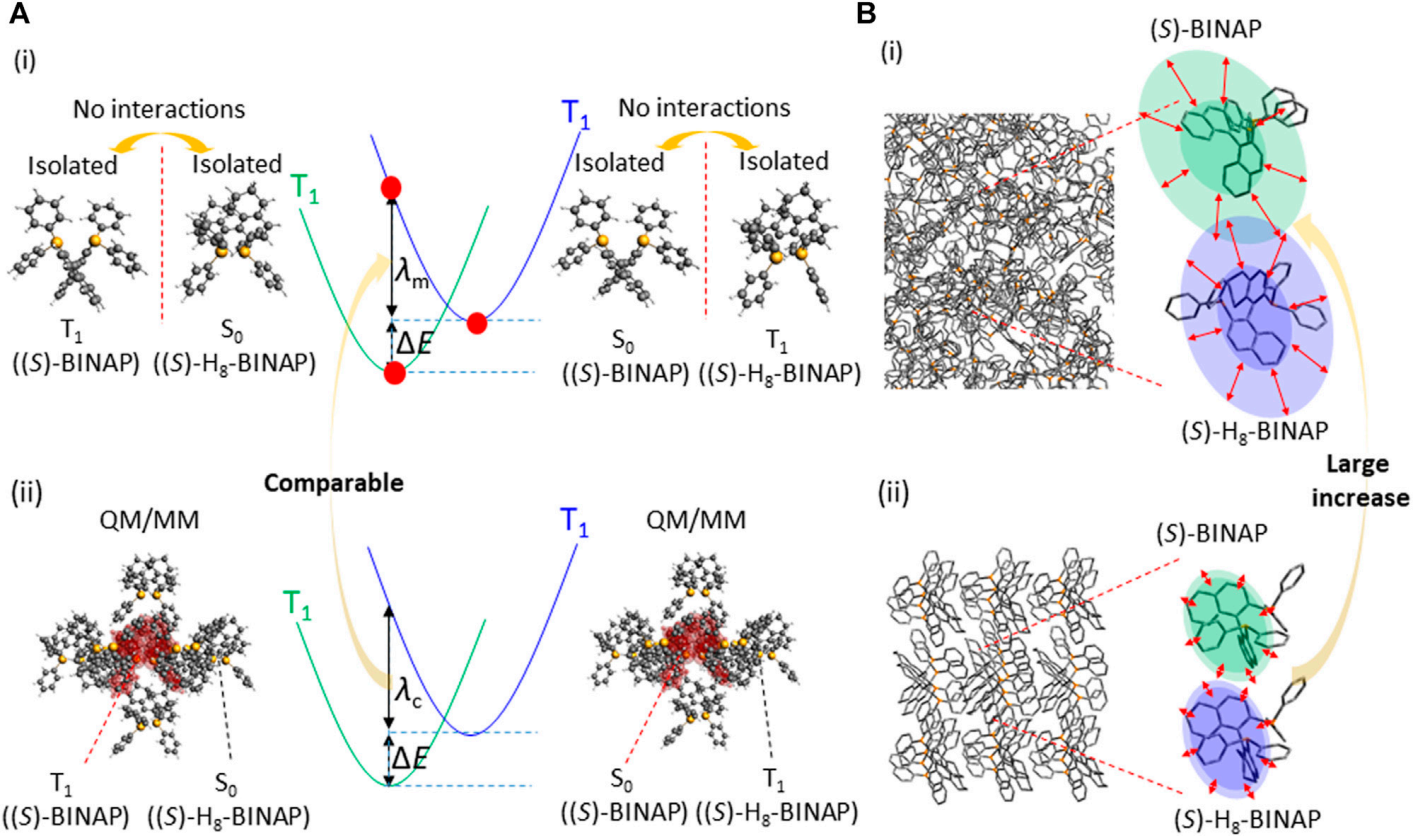
Physical behavior relating to *k*_q_(RT) except for Δ*E* from the perspective of intermolecular interactions between an (*S*)-BINAP guest and the (*S*)-H_8_-BINAP host molecules. **(A)** Reorganization energy for endothermic triplet–triplet energy transfer. (i) represents a case in which (*S*)-BINAP and (*S*)-H_8_-BINAP have no intermolecular interactions. (ii) represents a case in which a molecule in a crystalline lattice of (*S*)-H_8_-BINAP is replaced by (*S*)-BINAP. **(B)** The diffusion constant; (i) and (ii) represent the amorphous and crystalline states, respectively. The red arrows indicate different magnitudes of the diffusion constant at RT between the amorphous and crystalline states. (RT = room temperature; (*S*)-BINAP = (*S*)-(−)-2,2′-bis(diphenylphosphino)-1,1′-binaphthyl (*S*)-H_8_-BINAP = (*S*)-bis(diphenylphosphino)-5,5′,6,6′,7,7′,8,8′-octahydro-1,1′-binaphthyl).

If *λ* in the crystalline state is much larger than it is in the amorphous state due to more potential intermolecular interactions, the difference in the number of intermolecular interactions causes a large difference in *k*_q_(RT), even when Δ*E* is comparable. Under the condition that (*S*)-BINAP had intermolecular interactions with the (*S*)-H_8_-BINAP molecules in the crystalline (*S*)-H_8_-BINAP lattice (*λ*_c_), the reorganization energy with regard to the energy transfer from T_1_ of (*S*)-BINAP to T_1_ of (*S*)-H_8_-BINAP was calculated to enable further discussion of the contribution made by *λ* to *k*_q_(RT) (Figure 6A; (ii)). Initially, a molecule of (*S*)-H_8_-BINAP in the crystalline lattice of (*S*)-H_8_-BINAP was replaced by (*S*)-BINAP (Supplementary Figure S11A; (I)). The S_0_ and T_1_ geometries of the replaced (*S*)-BINAP were optimized without changing any of the coordinates of the (*S*)-H_8_-BINAP molecules around the (*S*)-BINAP (Supplementary Figure S11A, (II)). Subsequently, the S_0_ and T_1_ geometries of the (*S*)-H_8_-BINAP molecule in the crystalline lattice of (*S*)-H_8_-BINAP were optimized without changing any of the coordinates of the (*S*)-H_8_-BINAP molecules around the target (*S*)-H_8_-BINAP (Supplementary Figure S11B; (III)). The four geometries were used to determine *λ*_c_, and *λ*_c_ = 1.59 eV was calculated using the Amsterdam Modeling Suite (AMS) 2020 software package with the B3LYP functional and the TZP basis set (Supplementary Material, Section 4). The reorganization energy relating to the energy transfer from T_1_ of (*S*)-BINAP to T_1_ of (*S*)-H_8_-BINAP under the condition that (*S*)-BINAP and (*S*)-H_8_-BINAP had no intermolecular interactions (*λ*_m_) was also calculated (Figure 6A; (i) and Supplementary Figure S12). The S_0_ and T_1_ geometries were calculated for isolated molecules of (*S*)-BINAP and (*S*)-H_8_-BINAP. The four geometries were used to determine *λ*_m_, and *λ*_m_ = 1.84 eV was calculated using the AMS 2020 software package with the B3LYP functional and the TZP basis set (Supplementary Material, Section 4). Although a larger *λ* in the crystalline state had been logically considered because intermolecular interactions may cause more energy to coordinate change for reorganization, *λ*_c_ was slightly smaller than *λ*_m_. For example, the reorganization energy for charge transfer between molecules in a crystalline semiconductor is 0.1 eV (Liu et al., 2017). However, this is often less than that calculated for isolated molecules without any intermolecular interactions (Narushima et al., 2019). Therefore, the local rigidity in molecular solids may be hardly related to the reorganization energy for charge transfer. Therefore, Δ*E* and *λ* do not properly explain the large increase in *k*_q_(RT) in the amorphous, compared with the crystalline, 5 wt% (*S*)-BINAP-doped (*S*)-H_8_-BINAP.

Another potential reason to change *k*_q_(RT) independently of Δ*E* and *λ* may be *D*(RT). Diffusion depends on Brownian motion. It is a different physical phenomenon from the vibrations and coordination changes that take place in molecules during reorganization (Brown, 1828; Einstein, 1905). The theory underlying Brownian motion was established in the early 1900s (von Smoluchowski, 1906). Because molecules in an amorphous state generally have a large free volume compared with those in a crystalline state, the local diffusion of molecules is allowed, even when the solids are below the glass transition temperature (Horie and Mita, 1982; Horie et al., 1984). The *D*(RT) of a side group in a representative amorphous polymer—i.e., polymethylmethacrylate—was reported to have a magnitude of 10^−14^ cm^2^/s (Horie et al., 1984), which is less than the *D*(RT) of a general liquid molecule, i.e., 1/10^8^ (Monguzzi et al., 2013). The *D*(RT) in the amorphous state may be large compared with that in the crystalline state for solid materials at RT. To enable triplet–triplet energy transfer, two molecular orbitals must overlap. In the amorphous state, a large *D*(RT) increases the possibility of the overlap of two molecular orbitals enabling triplet–triplet energy transfer (Figure 6B; (i)). However, stronger intermolecular interactions decrease *D*(RT), reducing the possibility of orbital overlap (Figure 6B; (ii)). Molecular dynamics (MD) analysis indicates that the simulated *D*(RT) of (*S*)-H_8_-BINAP in the amorphous state is approximately 10 times larger than it is in the crystalline state (Supplementary Figure S13). The magnitude of the difference in the calculated *D*(RT) between the amorphous and crystalline states is comparable to that of the *k*_q_(RT) between those states. Thus, the similar increase of magnitude of the enhanced *D*(RT) of crystalline (*S*)-H_8_-BINAP solid compared with amorphous (*S*)-H_8_-BINAP solid was observed between optically estimated *D*(RT) and simulated *D*(RT). To date there has been no discussion of Δ*E*, *λ*, and *D*(RT) with regard to *k*_q_(RT). By investigating large differences in *k*_q_(RT) between two solid materials with comparable Δ*E* and *λ* values, we attempted to confirm that the control of *D*(RT) is also a dominant factor in increasing the triplet quenching caused by endothermic triplet energy transfer. Therefore, more detailed discussions about molecular diffusion in solid materials are essential. The contribution of *D*(RT) to *k*_q_(RT) has not been addressed in research into organic electronics. The contribution made by Δ*E* to *k*_q_(RT) has not been investigated in research on aggregated chromophores that generate *p*RTP, and the relationship between rigidity and vibrations and intermolecular interactions remains unclear. The controlled results reported in the present paper indicate that discussions about both Δ*E* and *D*(RT) are crucial for the intrinsic control of *k*_q_(RT) and the long-lived RT triplet state of heavy atom-free and/or metal-free solid materials.

## Conclusion

In the present study, a thermo-reversible phase change of an (*S*)-H_8_-BINAP host doped with (*S*)-BINAP was induced. The (*S*)-BINAP produced a *p*RTP spectrum that was comparable to the spectra of the (*S*)-H_8_-BINAP host in the two phases. The molecularly dispersed (*S*)-BINAP had a *Φ*_p_(RT) of 6.7% in the crystalline (*S*)-H_8_-BINAP host and a *Φ*_p_(RT) of 0.31% in the amorphous host. Detailed photophysical analyses indicated that the triplet generation of (*S*)-BINAP and the magnitude of the difference in *k*_q_(RT) between the (*S*)-BINAP guest and the (*S*)-H_8_-BINAP host both differed between the amorphous and crystalline states. This caused a large difference in the *Φ*_p_(RT) of (*S*)-BINAP between those states. Differing Δ*E*, *λ*, and *D*(RT) values were considered as a potential explanation for the difference in *k*_q_(RT) values between the amorphous and crystalline states. Comparable Δ*E* and *λ* values in both the amorphous and crystalline states indicated that the *D*(RT) is the reason for the large discrepancy in *k*_q_(RT). The large difference in the magnitude of *D*(RT) between the amorphous and crystalline states was supported by MD simulations. The suppression of vibrations by intermolecular interactions is often used to explain the decrease in nonradiative deactivation from T_1_. However, analysis of Δ*E*, *λ*, and *D*(RT) from all perspectives clarifies that the small *D*(RT) in the solid state makes a large contribution to the control of *k*_q_(RT), and intermolecular interactions could be used to suppress molecular diffusion in the solid state, thereby reducing the *k*_q_(RT). Because the significantly low rate of diffusion in molecular solids is also logically related to solid-state chemical reactions, analysis of slow local diffusion may be crucial to the control of chemical conversion in the solid state (Kubota et al., 2019). Because an accurate estimation of the small diffusion constant in molecular solids is difficult, the analysis introduced in the present paper is important for the construction of a variety of materials that allow long-lived RT triplet excitons, and for the control of electron transfer and chemical reactions in molecular solids.

## Data Availability

The datasets presented in this study can be found in online repositories. The names of the repository/repositories and accession number(s) can be found in the article/Supplementary Material.

## References

[B1] Adachi C. Kwong R. C. Djurovich P. Adamovich V. Baldo M. A. Thompson M. E. (2001). Endothermic Energy Transfer: A Mechanism for Generating Very Efficient High-Energy Phosphorescent Emission in Organic Materials. Appl. Phys. Lett. 79, 2082–2084. 10.1063/1.1400076

[B2] Bhattacharjee I. Hayashi K. Hirata S. (2021). Key of Suppressed Triplet Nonradiative Transition-dependent Chemical Backbone for Spatial Self-Tunable Afterglow. JACS Au 1, 945–954. 10.1021/jacsau.1c00132 34467341 PMC8395709

[B3] Bhattacharjee I. Hirata S. (2020). Highly Efficient Persistent Room‐Temperature Phosphorescence from Heavy Atom‐Free Molecules Triggered by Hidden Long Phosphorescent Antenna. Adv. Mater. 32, 2001348. 10.1002/adma.202001348 32596857

[B4] Brown R. (1828). XXVII. A Brief Account of Microscopical Observations Made in the Months of June, July and August 1827, on the Particles Contained in the Pollen of Plants; and on the General Existence of Active Molecules in Organic and Inorganic Bodies. Philosophical Mag. 4, 161–173. 10.1080/14786442808674769

[B5] Chakraborty A. Chakrabarty D. Seth D. Hazra P. Sarkar N. (2006). Photo-Induced Intermolecular Electron Transfer from Electron Donating Solvents to Coumarin Dyes in Bile Salt Aggregates: Role of Diffusion in Electron Transfer Reaction. Spectrochimica Acta A: Mol. Biomol. Spectrosc. 63, 594–602. 10.1016/j.saa.2005.06.006 16027032

[B6] Clapp D. B. (1939). The Phosphorescence of Tetraphenylmethane and Certain Related Substances. J. Am. Chem. Soc. 61, 523–524. 10.1021/ja01871a504

[B7] Closs G. L. Johnson M. D. Miller J. R. Piotrowiak P. (1989). A Connection between Intramolecular Long-Range Electron, Hole, and Triplet Energy Transfers. J. Am. Chem. Soc. 111, 3751–3753. 10.1021/ja00192a044

[B8] Deng Y. Zhao D. Chen X. Wang F. Song H. Shen D. (2013). Long Lifetime Pure Organic Phosphorescence Based on Water Soluble Carbon Dots. Chem. Commun. 49, 5751–5753. 10.1039/C3CC42600A 23685471

[B9] Dexter D. L. (1953). A Theory of Sensitized Luminescence in Solids. J. Chem. Phys. 21, 836–850. 10.1063/1.1699044

[B10] Einstein A. (1905). Über die von der molekularkinetischen Theorie der Wärme geforderte Bewegung von in ruhenden Flüssigkeiten suspendierten Teilchen. Ann. Phys. 322, 549–560. 10.1002/andp.19053220806

[B11] Fateminia S. M. A. Mao Z. Xu S. Yang Z. Chi Z. Liu B. (2017). Organic Nanocrystals with Bright Red Persistent Room-Temperature Phosphorescence for Biological Applications. Angew. Chem. Int. Ed. 56, 12160–12164. 10.1002/ange.20170594510.1002/anie.201705945 28771963

[B12] Fukuzumi S. Ohkubo K. Imahori H. Guldi D. M. (2003). Driving Force Dependence of Intermolecular Electron-Transfer Reactions of Fullerenes. Chem. Eur. J. 9, 1585–1593. 10.1002/chem.200390182 12658657

[B13] Hirata S. Bhattacharjee I. (2021). Vibrational Radiationless Transition from Triplet States of Chromophores at Room Temperature. J. Phys. Chem. A. 125, 885–894. 10.1021/acs.jpca.0c09410 33467853

[B14] Hirata S. Hara H. Bhattacharjee I. (2020). Phosphorescence Quenching of Heavy-atom-free Dopant Chromophores Triggered by Thermally Activated Triplet Exciton Diffusion of a Conjugated Crystalline Host. J. Phys. Chem. C 124, 25121–25132. 10.1021/acs.jpcc.0c07864

[B15] Hirata S. (2017). Recent Advances in Materials with Room-Temperature Phosphorescence: Photophysics for Triplet Exciton Stabilization. Adv. Opt. Mater. 5, 1700116. 10.1002/adom.201700116

[B16] Hirata S. Totani K. Zhang J. Yamashita T. Kaji H. Marder S. R. (2013). Efficient Persistent Room Temperature Phosphorescence in Organic Amorphous Materials under Ambient Conditions. Adv. Funct. Mater. 23, 3386–3397. 10.1002/adfm.201203706

[B17] Horie K. Mita I. (1982). Photochemistry in Polymer Solids. Decay of Benzophenone Phosphorescence in Poly(methyl Methacrylate). Chem. Phys. Lett. 93, 61–65. 10.1016/0009-2614(82)85056-2

[B18] Horie K. Morishita K. Mita I. (1984). Photochemistry in Polymer Solids. 3. Kinetics for Nonexponential Decay of Benzophenone Phosphorescence in Acrylic and Methacrylic Polymers. Macromolecules 17, 1746–1750. 10.1021/ma00139a020

[B19] Köhler A. Bässler H. (2011). What Controls Triplet Exciton Transfer in Organic Semiconductors? J. Mater. Chem. 21, 4003–4011. 10.1039/C0JM02886J

[B20] Kubota K. Pang Y. Miura A. Ito H. (2019). Redox Reactions of Small Organic Molecules Using Ball Milling and Piezoelectric Materials. Science 366, 1500–1504. 10.1126/science.aay8224 31857482

[B21] Kwon M. S. Yu Y. Coburn C. Phillips A. W. Chung K. Shanker A. (2015). Suppressing Molecular Motions for Enhanced Room-Temperature Phosphorescence of Metal-free Organic Materials. Nat. Commun. 6, 8947. 10.1038/ncomms9947 26626796 PMC4686823

[B22] Liu C. Huang K. Park W.-T. Li M. Yang T. Liu X. (2017). A Unified Understanding of Charge Transport in Organic Semiconductors: the Importance of Attenuated Delocalization for the Carriers. Mater. Horiz. 4, 608–618. 10.1039/C7MH00091J

[B23] Louis M. Thomas H. Gmelch M. Haft A. Fries F. Reineke S. (2019). Blue‐Light‐Absorbing Thin Films Showing Ultralong Room‐Temperature Phosphorescence. Adv. Mater. 31, 1807887. 10.1002/adma.201807887 30721550

[B24] Marcus R. A. (1956). On the Theory of Oxidation‐Reduction Reactions Involving Electron Transfer. I. J. Chem. Phys. 24, 966–978. 10.1063/1.1742723

[B25] Metz F. Friedrich S. Hohlneicher G. (1972). What Is the Leading Mechanism for the Nonradiative Decay of the Lowest Triplet State of Aromatic Hydrocarbons? Chem. Phys. Lett. 16, 353–358. 10.1016/0009-2614(72)80291-4

[B26] Metz F. (1973). Position-Dependent Deuterium Effect on Relative Rate Constants for ISC Processes in Aromatic Hydrocarbons. Chem. Phys. Lett. 22, 186–190. 10.1016/0009-2614(73)80567-6

[B27] Monguzzi A. Bianchi F. Bianchi A. Mauri M. Simonutti R. Ruffo R. (2013). High Efficiency Up-Converting Single Phase Elastomers for Photon Managing Applications. Adv. Energ. Mater. 3, 680–686. 10.1002/aenm.201200897

[B28] Narushima K. Kiyota Y. Mori T. Hirata S. Vacha M. (2019). Suppressed Triplet Exciton Diffusion Due to Small Orbital Overlap as a Key Design Factor for Ultralong-Lived Room-Temperature Phosphorescence in Molecular Crystals. Adv. Mater. 31, 1807268. 10.1002/adma.201807268 30633401

[B29] Notsuka N. Kabe R. Goushi K. Adachi C. (2017). Confinement of Long‐Lived Triplet Excitons in Organic Semiconducting Host-Guest Systems. Adv. Funct. Mater. 27, 1703902. 10.1002/adfm.201703902

[B30] Schlag E. W. Schneider S. Fischer S. F. (1971). Lifetimes in Excited States. Annu. Rev. Phys. Chem. 22, 465–526. 10.1146/annurev.pc.22.100171.002341

[B31] Totani K. Okada Y. Hirata S. Vacha M. Watanabe T. (2013). Thermoresponsive Persistent Phosphorescent Color Change Using Efficient Thermally Activated Reverse Energy Transfer with a Large Energy Difference. Adv. Opt. Mater. 1, 283–288. 10.1002/adom.201300013

[B32] von Smoluchowski M. (1906). Zur kinetischen Theorie der Brownschen Molekularbewegung und der Suspensionen. Ann. Phys. 326, 756–780. 10.1002/andp.19063261405

[B33] Zhang G. Chen J. Payne S. J. Kooi S. E. Demas J. N. Fraser C. L. (2007). Multi-Emissive Difluoroboron Dibenzoylmethane Polylactide Exhibiting Intense Fluorescence and Oxygen-Sensitive Room-Temperature Phosphorescence. J. Am. Chem. Soc. 129, 8942–8943. 10.1021/ja0720255 17608480

[B34] Zhang X. Xie T. Cui M. Yang L. Sun X. Jiang J. (2014). General Design Strategy for Aromatic Ketone-Based Single-Component Dual-Emissive Materials. ACS Appl. Mater. Inter. 6, 2279–2284. 10.1021/am405209w 24484404

[B35] Zhao W. He Z. Tang B. Z. (2020). Room-Temperature Phosphorescence from Organic Aggregates. Nat. Rev. Mater. 5, 869–885. 10.1038/s41578-020-0223-z

[B36] Zhen X. Tao Y. An Z. Chen P. Xu C. Chen R. (2017). Ultralong Phosphorescence of Water-Soluble Organic Nanoparticles for *In Vivo* Afterglow Imaging. Adv. Mater. 29, 1606665. 10.1002/adma.201606665 28657119

[B37] Zhou B. Yan D. (2019). Simultaneous Long‐Persistent Blue Luminescence and High Quantum Yield within 2D Organic-Metal Halide Perovskite Micro/Nanosheets. Angew. Chem. Int. Ed. 58, 15128–15135. 10.1002/anie.201909760 31441190

