# Supplementary material for: Thermo-Reversible Persistent Phosphorescence Modulation Reveals the Large Contribution Made by Rigidity to the Suppression of Endothermic Intermolecular Triplet Quenching
Source: Front Chem. 2021 Nov 16;9:788577. doi: 10.3389/fchem.2021.788577 (PMC8636281; doi:10.3389/fchem.2021.788577)
Supplement: Supplementary file 1 [file DataSheet1.PDF]

Supplementary Material

Thermo-reversible persistent phosphorescence modulation reveals the large contribution made by rigidity to the suppression of endothermic intermolecular triplet quenching

*Tomoya Kusama, Shuzo Hirata**

Department of Engineering Science, The University of Electro-Communications, 1-5-1, Chofugaoka, Chofu, Tokyo 182-8585, Japan

*Corresponding author. E-mail: shuzohirata@uec.ac.jp

This file includes

1. Preparation of Materials

2. General Optical Measurements

3. Measurement to Determine the Yield of the Intersystem Crossing of Molecularly Dispersed (*S*)-BINAP

4. Calculations

5. Supplementary Figures and Tables

1. Preparation of Materials

Commercially available (*S*)-H_8-BINAP solids (Sigma-Aldrich, St Louis, USA) are crystalline and produce green persistent room-temperature phosphorescence (*p*RTP) with the spectral shape shown in **Supplementary Figure 1A**. However, the green *p*RTP is not caused by (*S*)-H_8-BINAP because no *p*RTP was observed from crystals of (*S*)-H_8-BINAP after careful repeated column chromatography (silica gels: eluent hexane/dichloromethane = 7:3 vol) (**Supplementary Figure 1B**) (Hirata et al., **2020**). The green *p*RTP is caused by the (*S*)-BINAP contained in the (*S*)-H_8-BINAP solid host, because the same *p*RTP spectra and decay lifetime are observed when small amounts of (*S*)-BINAP are doped into highly purified crystalline (*S*)-H_8-BINAP without producing *p*RTP (**Supplementary Figure 1C**). ^{1}H nuclear magnetic resonance (^{1}H NMR) spectra before purification of (*S*)-H_8-BINAP (**Supplementary Figure 2A**) contain small peaks in the range 6.82−6.92 ppm, which correspond to the ^{1}H NMR spectra of (*S*)-BINAP (**Supplementary Figure 2B**). After the carefully repeating column chromatography, the small peaks corresponding to (*S*)-BINAP disappeared (**Supplementary Figure 2C**). (*S*)-H_8-BINAP is generally synthesized from (*S*)-BINAP. However, the reaction yield may be 100% and small amounts of (*S*)-BINAP may remain. Moreover, careful separation by chromatography is time-consuming. During recrystallization, however, the (*S*)-BINAP cannot be removed. This is probably because the size and structure of (*S*)-BINAP are very similar to those of (*S*)-H_8-BINAP. Repeated sublimation is not a satisfactory method of removing (*S*)-BINAP because the molecular weight of (*S*)-BINAP is comparable to that of (*S*)-H_8-BINAP. In aromatic crystals, heat-driven triplet migration is often significant (Hirata et al. **2020**), even when the concentration of the phosphorescence triplet traps is low (less than 0.01 wt%). Therefore, *p*RTP is produced by tiny chromophores that become deep triplet traps. Please note that the experiments described herein were carried out using (*S*)-H_8-BINAP solids that did not produce *p*RTP.

(*S*)-BINAP powder was dissolved in molten (*S*)-H_8-BINAP at 220°C. The molten materials were placed on a quartz substrate on a hotplate at 250°C, and the substrate was quenched to room temperature (RT) to prepare an amorphous 5 wt% (*S*)-BINAP-doped (*S*)-H_8-BINAP film. The amorphous film on the quartz substrate was heated at 165°C for 30 min to prepare a crystalline 5 wt% (*S*)-BINAP-doped (*S*)-H_8-BINAP film. Amorphous 5 wt% (*S*)-BINAP-doped (*S*)-H_8-BINAP was prepared again by heating the substrate at 250°C and quenching it to RT. To prepare a 0.3 wt% (*S*)-BINAP-doped amorphous β-estradiol film, 1 mg of (*S*)-BINAP and 333 mg of β-estradiol (Tokyo Chemical Industry, Japan) were heated on a quartz substrate on a hotplate at 250°C. After dissolving the (*S*)-BINAP in the molten β-estradiol, the material on the quartz substrate was quenched to RT. All procedures were performed under ambient conditions. Differential scanning calorimetry (DSC) characteristics of the purified (*S*)-H_8-BINAP powder and (*S*)-BINAP powder are shown in **Supplementary Figure 3**.

2. General Physical and Optical Measurements

DSC characteristics of materials are measured using an Ultima III automated multipurpose X-ray diffractometer (Rigaku, Tokyo, Japan). Absorption spectra of the solutions and films were obtained using a V-760 absorption spectrometer (Jasco, Tokyo, Japan). Time-dependent emission intensities and phosphorescence decay characteristics were determined using a PMA-12 photonic multichannel analyzer (Hamamatsu Photonics, Shizuoka, Japan). Monochromatic light from the excitation unit of a

FP-8300 fluorimeter (Jasco) was used for excitation. The RT steady-state emission yields were measured using a C9920-02G absolute photoluminescence quantum yield spectrometer (Hamamatsu Photonics). The contribution made by *p*RTP to the steady-state emission yield was determined by comparing the areas under the curves of the steady-state emission and *p*RTP spectra immediately after ceasing excitation. Phosphorescence lifetimes were measured using a PMA-12 photonic multichannel analyzer (Hamamatsu Photonics). For optical measurements, the temperatures of the samples were adjusted using an Optistat DN-V cryostat (Oxford Instruments, United Kingdom).

3. Determination of the Yield of the Intersystem Crossing of Molecularly Dispersed (*S*)-BINAP

The yield of the intersystem crossing from the lowest singlet excited state (S_1) to the triplet states (Φ_{isc}) of the molecularly dispersed (*S*)-BINAP was determined using previously reported transient absorption techniques (Bhattacharjee et al. **2021**). A picoTAS sub-nanosecond transient absorption spectrophotometer (Unisoku, Osaka, Japan) used to obtain the transient absorption measurements, and secondary harmonic generation from a 532 nm Q-switched microchip laser (PNV-M02010-130; Teem Photonics, Meylan, France) was used for excitation.

The Φ_{isc} of the molecularly dispersed (*S*)-BINAP was obtained using the following sample and reference solutions. The sample solution comprised (*S*)-BINAP as the donor and 1×10^{-3} M β-carotene as the acceptor in tetrahydrofuran (THF). The reference solution comprised bis(2,4-difluorophenylpyridinato)-tetrakis(1-pyrazolyl)borate iridium(III) (FIr6) as the donor and 1×10^{-3} M β-carotene as the acceptor in THF. The solutions were degassed three times using the freeze-dry pump method, and sealed immediately before obtaining the measurements. Absorbance at 266 nm caused by (*S*)-BINAP or FIr6 was set to 5.0 in a 1 mm thick quartz cell. Triplet–triplet absorption after excitation at 266 nm by the sample and reference solutions (**Supplementary Figure 6A** and **Figure 6B**) indicated that the T_1 generated in (*S*)-BINAP or FIr6 had been transferred to the T_1 of β-carotene, and a large absorption peak at 500–550 nm confirmed triplet–triplet absorption in β-carotene. The transient absorption decay characteristics at 530 nm of the degassed sample and reference solutions were determined (**Supplementary Figure 6C**). In **Supplementary Figure 6C**, the fine red lines are the fitting lines of equation: $A(1 - \exp(- t/\tau_1))\exp(- t/\tau_2)+B\exp(- t/\tau_1)$, where t is the time after irradiation of the excitation pulse at 266 nm. The values of A, B, and τ_1 of the sample and reference solutions were determined by fitting, and are represented in **Supplementary Table 2**. The triplet–triplet absorption decay characteristics of (*S*)-BINAP in the absence of β-carotene in THF (**Supplementary Figure 6D**; (i)), and the triplet–triplet absorption decay characteristics of FIr6 in the absence of β-carotene in THF (**Supplementary Figure 6D**; (ii)) were also measured to determine the average lifetime of the triplet states of the donors (τ_0). The efficiency of the energy transfer from T_1 of the donor to T_1 of β-carotene in THF (Φ_{TT}) was calculated using $\Phi_{TT} = (\tau_0 - \tau_1)/\tau_0$.

The Φ_{isc} of (*S*)-BINAP in THF was determined based on $\Phi_{isc} = \alpha A/\Phi_{TT}$, where α is the constant value when the optics and excitation power at 266 nm are the same in the sample solution and the reference solution. Because the value of Φ_{isc} of FIr6 as a donor in the reference solution was 1, the value of α was determined using $\Phi_{isc} = 1$, A, and the Φ_{TT} value of the reference solution. Because A and Φ_{TT} of the sample solution were determined using the same optics and excitation conditions used in the reference solution, the value of Φ_{isc} of (*S*)-BINAP in benzene was determined by substituting the values of α, A, and Φ_{TT} of the sample solution into $\Phi_{isc} = \alpha A/\Phi_{TT}$.

4. Calculations

To calculate the T_1−S_0 transition energy and the k_p of the isolated (*S*)-BINAP (**Supplementary Figure 7**), the T_1 geometry of the isolated (*S*)-BINAP was calculated using the Gaussin09 software package based on density functional theory (DFT) with the B3LYP functional and the 6-31G(d) basis set. The geometries were used to calculate the T_1−S_0 transition energy of (*S*)-BINAP using the Amsterdam Density Functional Theory (ADF) 2018 software package with the PBE0 functional and the TZP basis set. The spin–orbit coupling operator within the zero-order regular approximation was $\hat{H}_{so}$. The spin–orbit coupling between the nth order triplet excited state (T_n) and the nth order singlet excited state (S_n) ($\langle S_n|\hat{H}_{so}|T_n\rangle$) was treated as a perturbation based on the scalar relativistic orbitals with the PBE0 functional and TZP basis sets. The scalar relativistic–time-dependent DFT calculations included 10 singlet + 10 triplet excitations, which were used as the basis for the perturbative expansions to calculate the T_1−S_0 transition energy, and $k_p = 0.22\ s^{-1}$.

To calculate the $\sum|\langle T_1|H_{SO}|S_0\rangle/\partial Q_p|^2 P(T)$ of the isolated (*S*)-BINAP, the mass weighted normal coordinates, which depend on the vibrations at T_1 (Q_p), were calculated using the Gaussin09 software package with the B3LYP functional and the 6-31G(d) basis set. $\langle S_0|\hat{H}_{so}|T_1\rangle$, which depends on Q_p [$\langle T_1|\hat{H}_{so}|S_0\rangle_{Q_p}$], was calculated using the ADF2018 software package from Q_p = - 1.0 to Q_p = + 1.0 with the PBE0 functional and the TZP basis set. The scalar relativistic–time-dependent DFT calculations included 1 singlet + 1 triplet excitations, which were used as the basis for the perturbative expansions to calculate $\sum|\langle T_1|H_{SO}|S_0\rangle/\partial Q_p|^2 P(T)$. Details of the procedure have been reported (Hirata and Bhattacharjee, **2021**).

To investigate the reorganization energy for the triplet–triplet energy transfer of molecules assuming no intermolecular interactions, the S_0 geometry of the isolated (*S*)-BINAP was calculated using the Amsterdam Modeling Suite (AMS) 2020 software package with the B3LYP functional and the TZP basis set. The total energy of (*S*)-BINAP with the S_0 electronic structure ((i) in **Supplementary Figure 12A**) and the total energy of (*S*)-BINAP with the T_1 electronic structure ((ii) in **Supplementary Figure 12A**) were calculated using the S_0 geometry. The T_1 geometry of the isolated (*S*)-BINAP was calculated with the B3LYP functional and the TZP basis set. The total energy of (*S*)-BINAP with the S_0 electronic structure ((iii) in **Supplementary Figure 12A**) and the total energy of (*S*)-BINAP with the T_1 electronic structure ((iv) in **Supplementary Figure 12A**) were also calculated using the T_1 geometry. The reorganization energy for the T_1-S_0 transition of the isolated (*S*)-BINAP ($\lambda_1 + \lambda_2$) was calculated using the four energies ((i)−(iv) in **Supplementary Figure 12A**). The reorganization energy for the S_0-T_1 transition of the isolated (*S*)-H_8-BINAP ($\lambda_3 + \lambda_4$) was calculated using the same procedure (**Supplementary Figure 12B**). The reorganization energy for the energy transfer from the T_1 of (*S*)-BINAP to the T_1 of (*S*)-H_8-BINAP under the condition that (*S*)-BINAP and (*S*)-H_8-BINAP had no intermolecular interactions (λ_m) was determined as the total energy of $\lambda_1 + \lambda_2$ and $\lambda_3 + \lambda_4$.

To investigate the reorganization energy for the triplet–triplet energy transfer of molecules with intermolecular interactions, a (*S*)-H_8-BINAP molecule in the crystalline lattice of (*S*)-H_8-BINAP was replaced by a (*S*)-BINAP molecule (**Supplementary Figure 11A**; (I)). The S_0 and T_1 geometries of the replaced (*S*)-BINAP were optimized without changing any of the coordinates of the (*S*)-H_8-BINAP molecules around the (*S*)-BINAP using the AMS 2020 software package with the B3LYP functional and the TZP basis set (**Supplementary Figure 11A**, (II)). The total energy of geometry (A) with the S_0 electronic structure of (*S*)-BINAP ((i) in **Supplementary Figure 11A**) and the total energy of geometry (A) with the T_1 electronic structure of (*S*)-BINAP ((ii) in **Supplementary Figure 11A**) were

calculated. The total energy of geometry (B) with the S_0 electronic structure of (*S*)-BINAP ((iii) in **Supplementary Figure 11A**) and the total energy of geometry (B) with the T_1 electronic structure of (*S*)-BINAP ((iv) in **Supplementary Figure 11A**) were also calculated. The reorganization energy for the S_0−T_1 transition of (*S*)-BINAP in the crystalline lattice of (*S*)-H_8-BINAP ($\lambda_1' + \lambda_2'$) was calculated using the four energies ((i)−(iv) in **Supplementary Figure 11A**). The S_0 and T_1 geometries at the center of (*S*)-H_8-BINAP in the crystalline lattice of (*S*)-H_8-BINAP were optimized without changing any of the coordinates of the (*S*)-H_8-BINAP molecules around the center of (*S*)-H_8-BINAP (**Supplementary Figure 11B**, (III)). The total energy of geometry (C) with the S_0 electronic structure at the center of (*S*)-H_8-BINAP ((i) in **Supplementary Figure 11B**) and the total energy of geometry (C) with the T_1 electronic structure at the center (*S*)-H_8-BINAP ((ii) in **Supplementary Figure 11B**) were calculated. The total energy of geometry (D) with the S_0 electronic structure at the center (*S*)-H_8-BINAP ((iii) in **Supplementary Figure 11B**) and the total energy of geometry (D) with the T_1 electronic structure at the center (*S*)-H_8-BINAP ((iv) in **Supplementary Figure 11B**) were also calculated. The reorganization energy for the S_0-T_1 transition of a molecule of (*S*)-BINAP in the crystalline lattice of (*S*)-H_8-BINAP ($\lambda_3' + \lambda_4'$) was calculated using the four energies ((i)−(iv) in **Supplementary Figure 11B**). The reorganization energy for the energy transfer from the T_1 of (*S*)-BINAP to the T_1 of (*S*)-H_8-BINAP under the condition that (*S*)-BINAP had intermolecular interactions with (*S*)-H_8-BINAP molecules in the crystalline (*S*)-H_8-BINAP lattice (λ_c) was determined as the total energy of $\lambda_1' + \lambda_2'$ and $\lambda_3' + \lambda_4'$.

The diffusion constant at RT (*D*(RT)) was estimated using the Gromax calculation with the General Amber Force Field. To determine the amorphous structure of solid (*S*)-H_8-BINAP, the S_0 geometry of (*S*)-H_8-BINAP was optimized using DFT (Gaussian09/B3LYP/6-31G(d)). Next, 100 molecules with the optimized S_0 geometry were set as an initial condition. After appropriate equilibration (**Supplementary Figure 13A**), the molecular dynamics simulation was performed for 20 ps at 300K and 1 bar to determine the *D*(RT) of the amorphous (*S*)-H_8-BINAP solid (**Supplementary Figure 13C**). To determine the crystalline structure of solid (*S*)-H_8-BINAP, 3×3×3 crystalline lattice structures, corresponding to 108 molecules, were set as an initial condition. After appropriate equilibration using the coordinates (**Supplementary Figure 13B**), the MD simulation was performed for 20 ps at 300K and 1 bar to determine the *D*(RT) of the crystalline (*S*)-H_8-BINAP solid (**Supplementary Figure 13C**).

5. Supplementary Figures and Tables

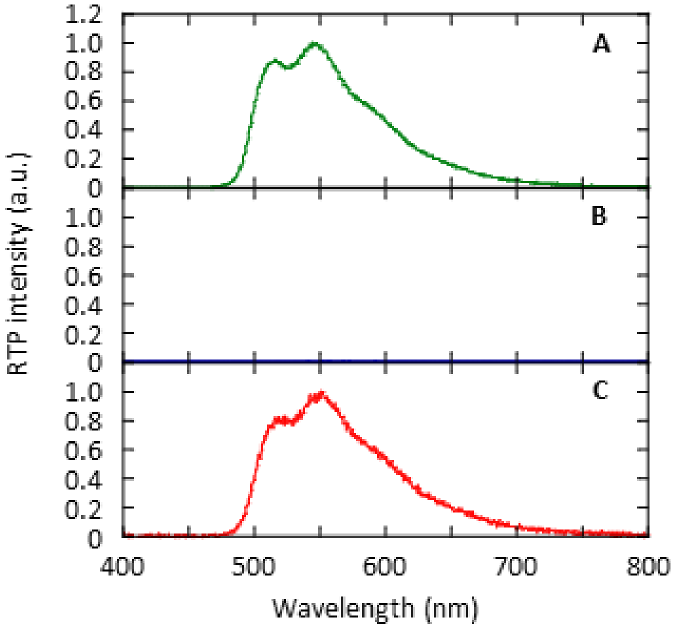

Supplementary Figure 1. *p*RTP caused by impurities in commercially available (*S*)-H_8-BINAP. **(A)** *p*RTP spectra of commercially available (*S*)-H_8-BINAP crystalline solids after ceasing excitation at 330 nm. **(B)** No afterglow emission after ceasing excitation at 330 nm in crystalline (*S*)-H_8-BINAP solids after purification by repeated chromatography. **(C)** *p*RTP spectra of crystalline 1 wt% (*S*)-BINAP-doped pure (*S*)-H_8-BINAP solids after ceasing excitation at 330 nm. (*p*RTP = persistent room-temperature phosphorescence; (*S*)-H_8-BINAP = (*S*)-bis(diphenylphosphino)-5,5′,6,6′,7,7′,8,8′-octahydro-1,1′-binaphthyl; (*S*)-BINAP = (*S*)-(−)-2,2′-bis(diphenylphosphino)-1,1′-binaphthyl).

A

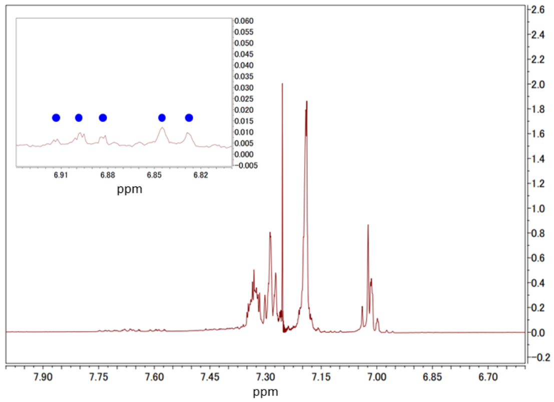

B

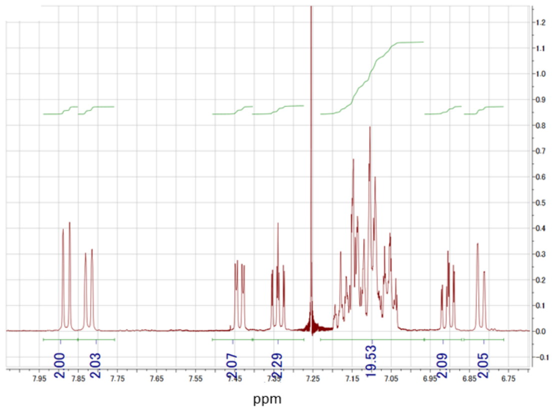

C

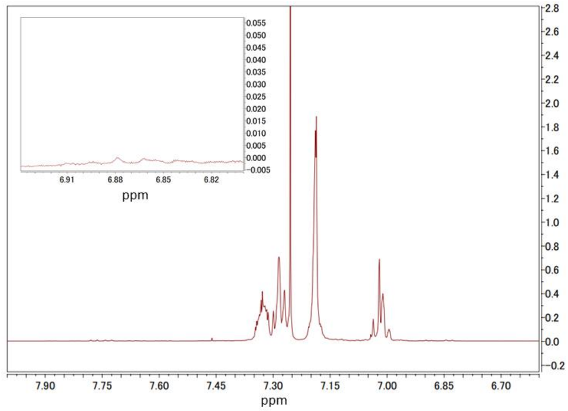

Supplementary Figure 2. 1H NMR spectra in $CDCl_3$. **(A)** (*S*)-H_8-BINAP before purification. **(B)** (*S*)-BINAP before purification. **(C)** (*S*)-H_8-BINAP after purification. ((*S*)-BINAP = (*S*)-(−)-2,2′-bis(diphenylphosphino)-1,1′-binaphthyl; (*S*)-H_8-BINAP = (*S*)-bis(diphenylphosphino)-5,5′,6,6′,7,7′,8,8′-octahydro-1,1′-binaphthyl).

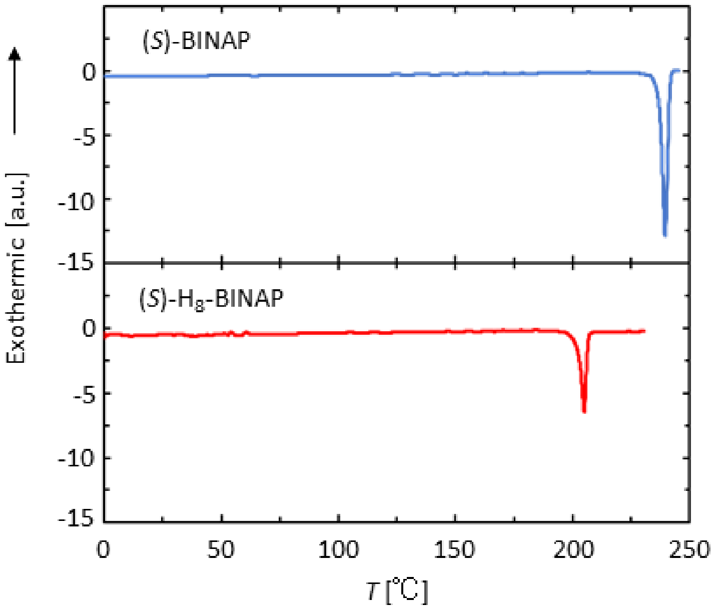

Supplementary Figure 3. DSC characteristics of (*S*)-BINAP powder (top) and purified (*S*)-H_8-BINAP powder (bottom). ((*S*)-BINAP = (*S*)-(−)-2,2′-bis(diphenylphosphino)-1,1′-binaphthyl; (*S*)-H_8-BINAP = (*S*)-bis(diphenylphosphino)-5,5′,6,6′,7,7′,8,8′-octahydro-1,1′-binaphthyl).

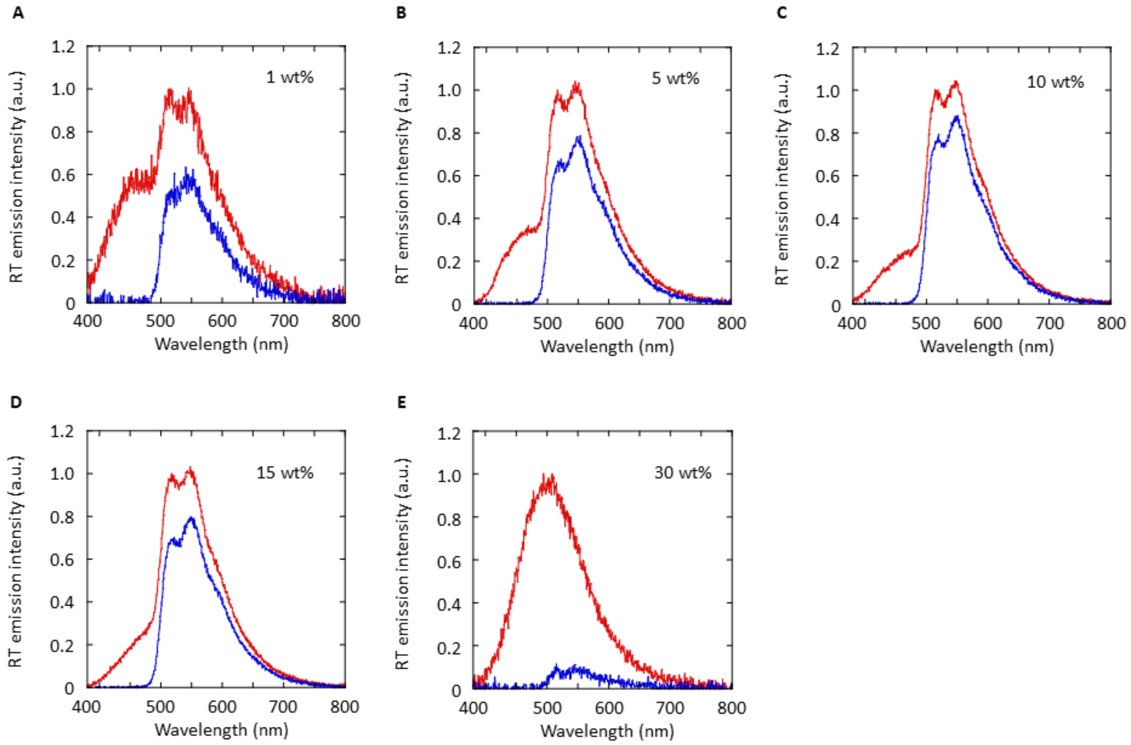

Supplementary Figure 4. Relative steady-state RT emission spectral intensity (red) and afterglow RT emission spectral intensity soon after ceasing excitation (blue) of (*S*)-H_8-BINAP crystals doped with different concentration of (*S*)-BINAP. **(A)** 1 wt%, **(B)** 5 wt%, **(C)** 10 wt%, **(D)** 15 wt%, and **(E)** 30 wt%, Excitation wavelength is 330 nm. (RT = room temperature; (*S*)-BINAP = (*S*)-(−)-2,2′-bis(diphenylphosphino)-1,1′-binaphthyl).

Supplementary Table 1. Summary of persistent RTP yield and lifetime of (*S*)-H_8-BINAP crystalline materials doped with different concentration of (*S*)-BINAP. (RTP = room-temperature phosphorescence; (*S*)-BINAP = (*S*)-(−)-2,2′-bis(diphenylphosphino)-1,1′-binaphthyl; (*S*)-H_8-BINAP = (*S*)-bis(diphenylphosphino)-5,5′,6,6′,7,7′,8,8′-octahydro-1,1′-binaphthyl).

Concentration (wt%)	Φ_f(RT) (%)	Φ_p(RT) (%)
1	3.1	2.0
5	3.1	6.7
10	2.6	5.0
15	2.7	6.0
30	3.7	0.27

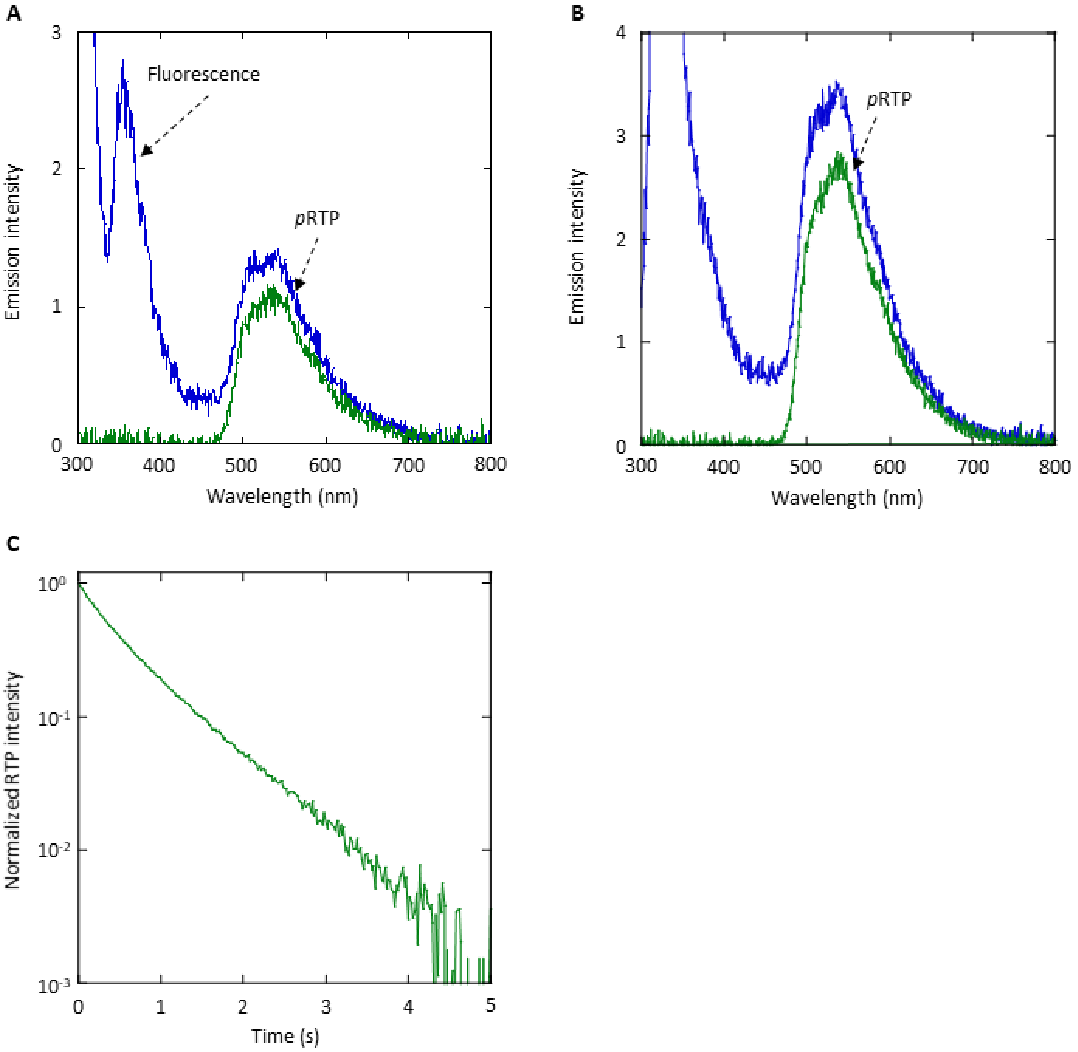

Supplementary Figure 5. RT Emission characteristics of 0.3 wt% (*S*)-BINAP-doped amorphous *β*-estradiol. **(A)** Steady-state RT emission spectra obtained by exposure to 300 nm radiation (blue) and afterglow RT emission spectra obtained immediately after ceasing excitation (green). **(B)** Steady-state RT emission spectra obtained by exposure to 330 nm radiation (blue) and afterglow RT emission spectra obtained immediately after ceasing excitation (green). **(C)** Afterglow RT emission decay characteristics. (RT = room temperature; RTP = room-temperature phosphorescence; (*S*)-BINAP = (*S*)-(−)-2,2′-bis(diphenylphosphino)-1,1′-binaphthyl).

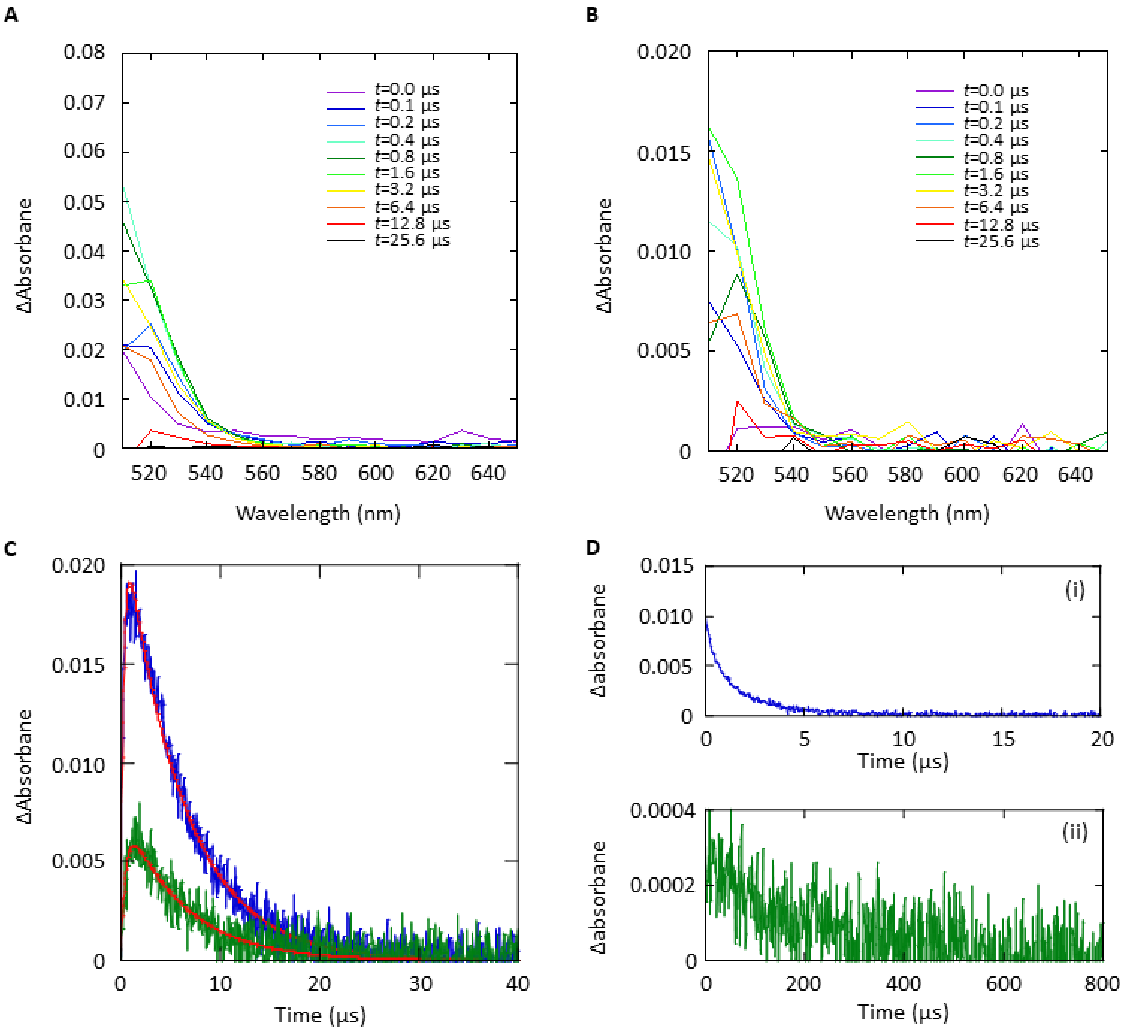

Supplementary Figure 6. Transient absorption characteristics of molecularly dispersed (*S*)-BINAP for the determination of Φ_{isc}. The excitation wavelength was 266 nm. **(A)** Spectral change in transient absorption of a degassed solution comprising FIr6 and 1 × 10^{-3} M *β*-carotene in THF. **(B)** Spectral change in transient absorption of a degassed solution comprising (*S*)-BINAP and 1 × 10^{-3} M *β*-carotene in THF. **(C)** Transient absorption decay at 530 nm of a degassed solution comprising FIr6 and 1 × 10^{-3} M *β*-carotene in THF (blue) and a degassed solution comprising (*S*)-BINAP and 1 × 10^{-3} M *β*-carotene in THF (green). **(D)** Transient absorption decay at 530 nm of a degassed solution comprising FIr6 in THF (upper) and transient absorption decay at 530 nm of a degassed solution comprising (*S*)-BINAP in THF (lower). For the solutions used in **(A)-(D)**, absorbance at 266 nm caused by FIr6 or (*S*)-BINAP in a 1 mm thick quartz cell was set to 5.0. ((*S*)-BINAP = (*S*)-(−)-2,2′-bis(diphenylphosphino)-1,1′-binaphthyl; THF = tetrahydrofuran).

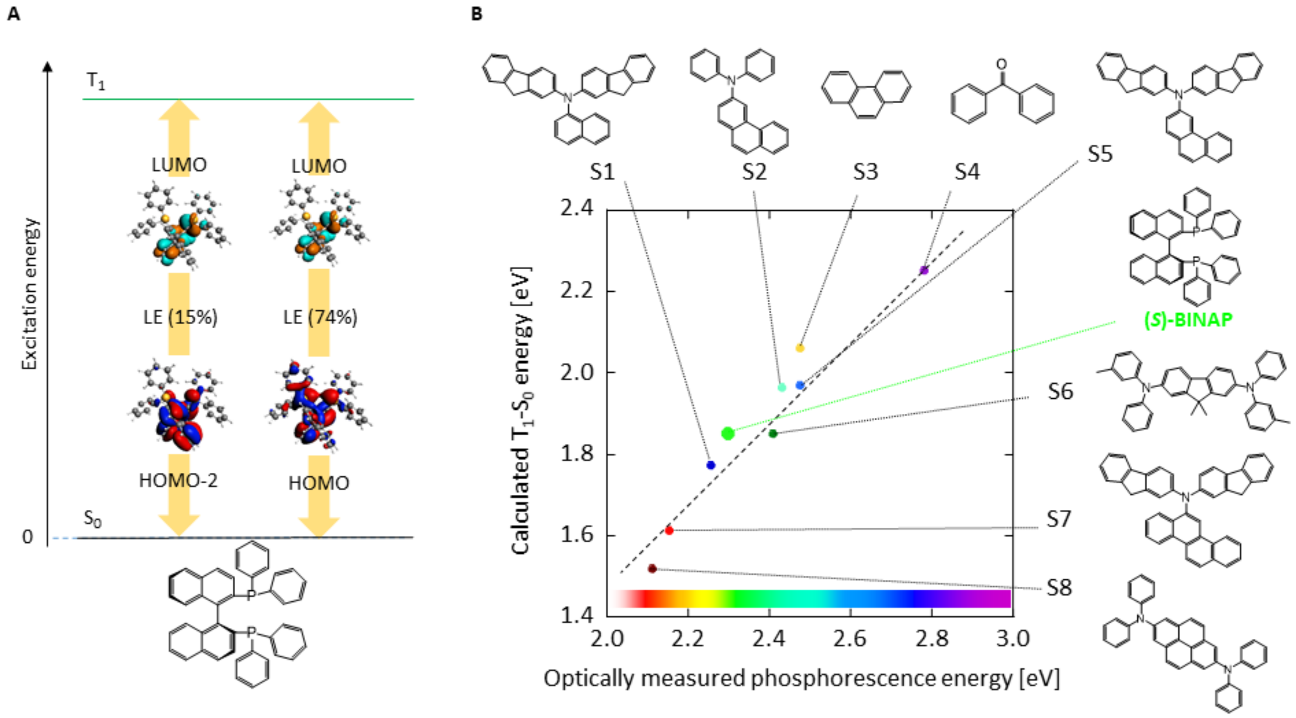

Supplementary Figure 7. Molecular orbitals relating to the T_1−S_0 transition and analysis of the T_1-S_0 transition energy. **(A)** Molecular orbitals relating to the T_1−S_0 transition of (*S*)-BINAP. **(B)** Comparison between the calculated T_1−S_0 transition energy and the optically measured T_1−S_0 transition energy. In **(A)** and **(B)**, the compounds were optimized to T_1 geometry using DFT (Gaussian09/B3LYP/6-31G(d)). Using the optimized geometries, the T_1-S_0 transition energies and molecular orbitals relating to the T_1−S_0 transition were calculated using the Amsterdam DFT software package (the ADF2018 package) with the PBE0 functional and the TZP basis set. In **(B)**, the optically estimated T_1−S_0 transition energies were determined from the peak wavelength of RTP. For chromophores S1−S6, the optical data were obtained from Hirata and Bhattacharjee, **2021**. For chromophores S7 and S8, the optical data were obtained from Bhattacharjee and Hirata, **2020** and Kamatsuki et al., **2020**, 11, 8675−8681, respectively. ((*S*)-BINAP = (*S*)-(−)-2,2′-bis(diphenylphosphino)-1,1′-binaphthyl; RTP = room-temperature phosphorescence).

Supplementary Table 2. Parameters used to determine the Φ_{isc} of molecularly dispersed (*S*)-(−)-2,2′-bis(diphenylphosphino)-1,1′-binaphthyl ((*S*)-BINAP).

Role of solution	Triplet sensitizer	The concentration of *β*-carotene (M)	*A*	*B*	τ_1 (μs)	τ_0 (μs)	Φ_{TT}	Φ_{isc}
Sample	(*S*)-BINAP	1×10^{-3}	0.00783	0.00042	0.525	504	1.00	0.29
Reference	FIr6	1×10^{-3}	0.0233	0.0020	0.299	2.28	0.868	1.00

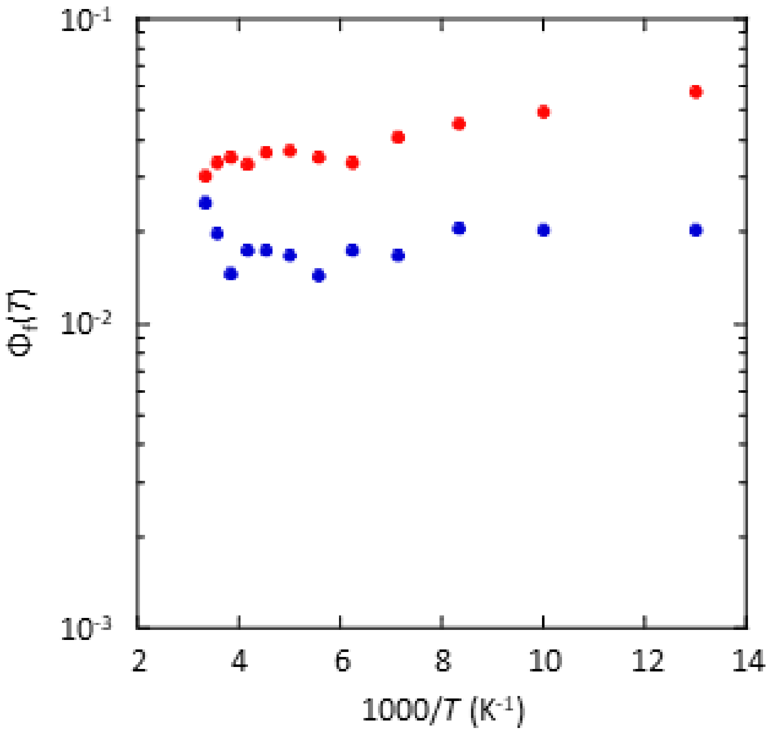

Supplementary Figure 8. Temperature dependence of the fluorescence yield (Φ_f) of amorphous (blue) and crystalline (red) 5 wt% (*S*)-BINAP-doped (*S*)-H_8-BINAP. The excitation wavelength was 330 nm. ((*S*)-BINAP = (*S*)-(−)-2,2′-bis(diphenylphosphino)-1,1′-binaphthyl; (*S*)-H_8-BINAP = (*S*)-bis(diphenylphosphino)-5,5′,6,6′,7,7′,8,8′-octahydro-1,1′-binaphthyl).

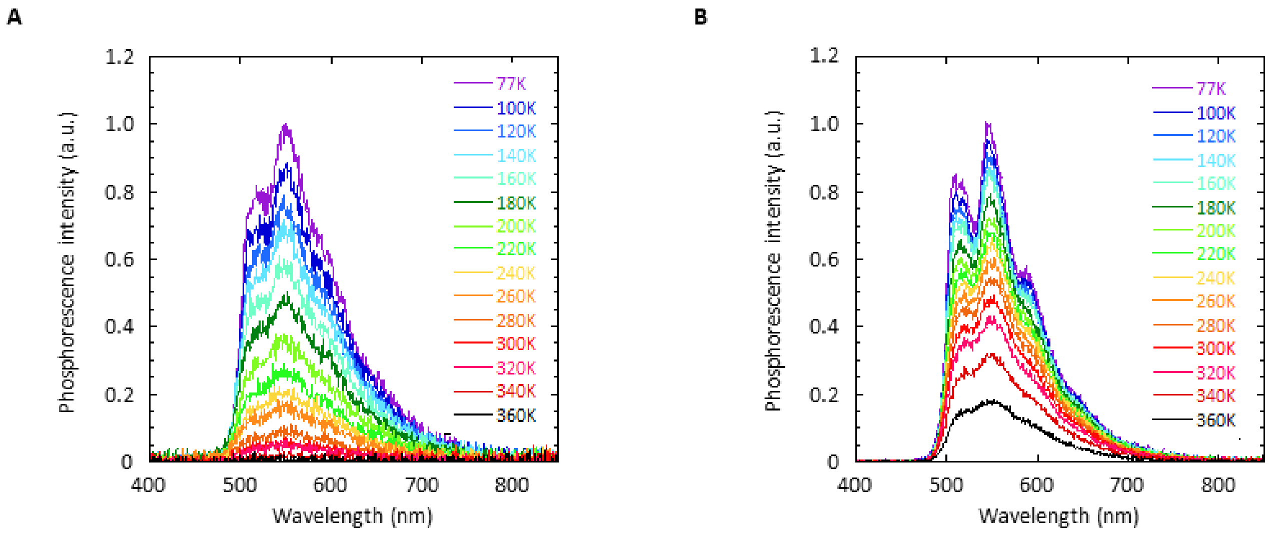

Supplementary Figure 9. Temperature dependence of phosphorescence intensity. **(A)** Amorphous 5 wt% (*S*)-BINAP-doped (*S*)-H_8-BINAP. **(B)** Crystalline 5 wt% (*S*)-BINAP-doped (*S*)-H_8-BINAP. The excitation wavelength was 330 nm. ((S)-BINAP = (*S*)-(−)-2,2′-bis(diphenylphosphino)-1,1′-binaphthyl; (*S*)-H_8-BINAP = (*S*)-bis(diphenylphosphino)-5,5′,6,6′,7,7′,8,8′-octahydro-1,1′-binaphthyl).

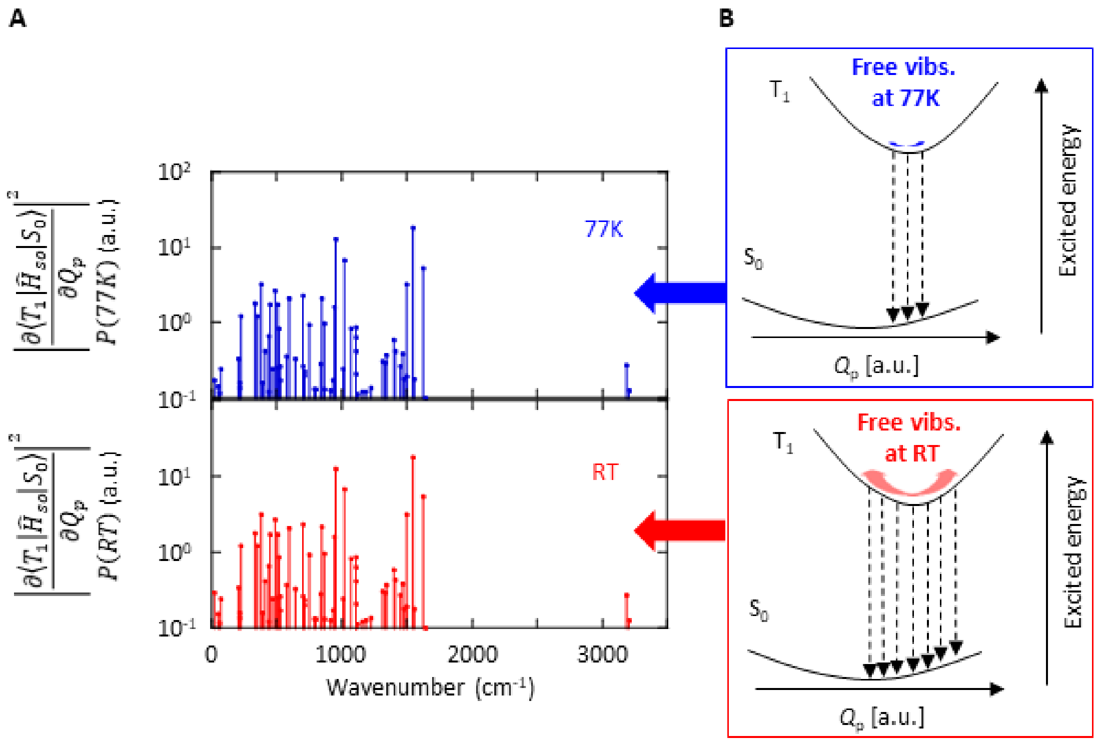

Supplementary Figure 10. Theoretical investigation of the Franck–Condon radiationless T_1–S_0 transition of molecularly dispersed (*S*)-BINAP. **(A)** Spectra of $|\langle T_1|H_{SO}|S_0\rangle/\partial Q_p|^2$(77K) (upper) and $|\langle T_1|H_{SO}|S_0\rangle/\partial Q_p|^2 P$(RT) (lower). **(B)** Illustrations explaining the Franck–Condon radiationless T_1–S_0 transition at 77K (upper) and RT (lower). In **(A)**, the integration of the $|\langle T_1|H_{SO}|S_0\rangle/\partial Q_p|^2$(77K) spectra and the integration of the $|\langle T_1|H_{SO}|S_0\rangle/\partial Q_p|^2$(RT) spectra hardly changed. This indicates that the k_{nr}(RT) of the molecularly dispersed (*S*)-BINAP hardly increased as the temperature increased from 77K to RT. ((*S*)-BINAP = (S)-(−)-2,2′-bis(diphenylphosphino)-1,1′-binaphthyl; RT = room temperature).

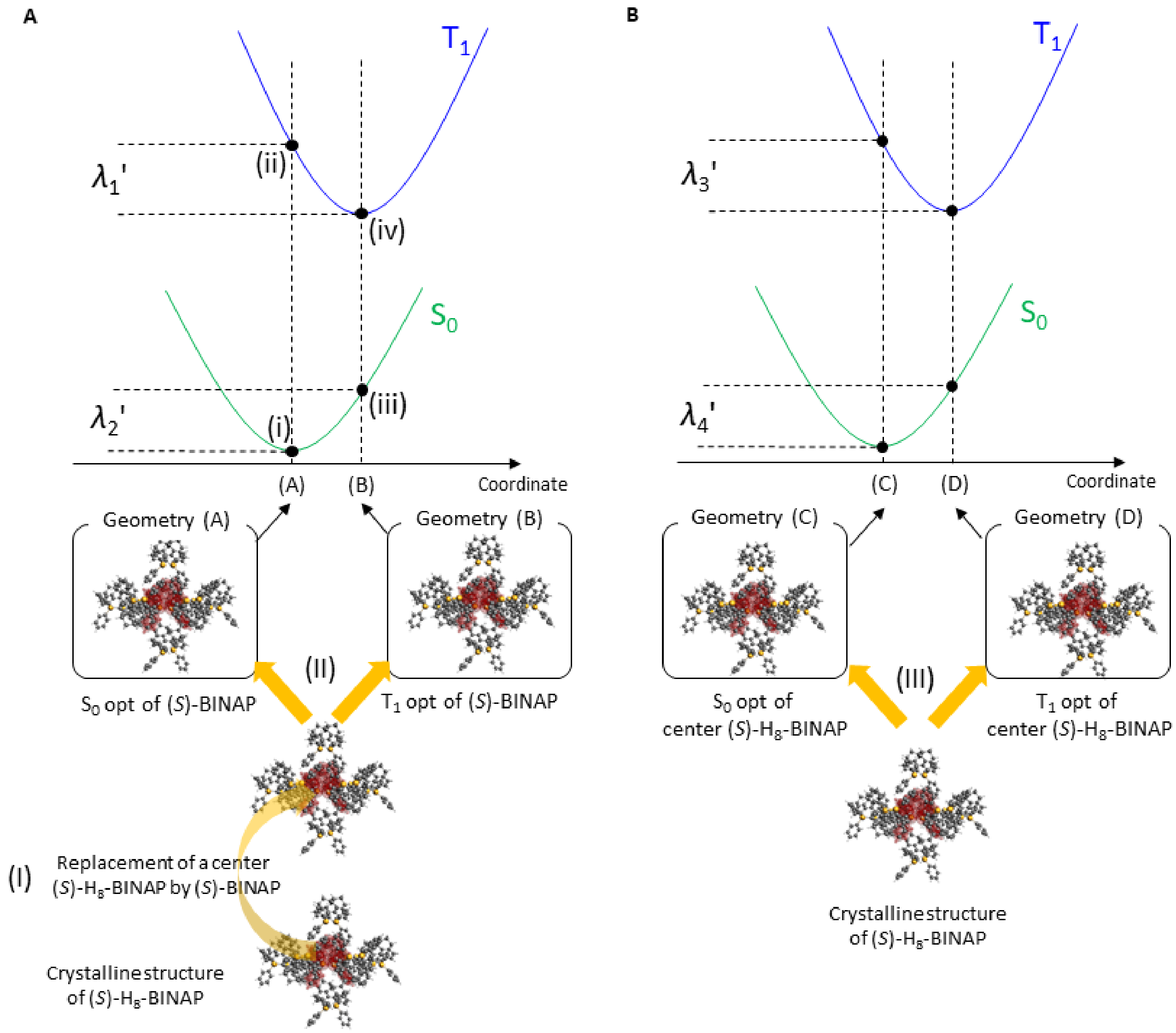

Supplementary Figure 11. Schematic of the procedure for calculating the reorganization energy of the energy transfer from the T_1 of (*S*)-BINAP to the T_1 of (*S*)-H_8-BINAP with the condition that (*S*)-BINAP has intermolecular interactions with (*S*)-H_8-BINAP molecules in the crystalline (*S*)-H_8-BINAP lattice. **(A)** The reorganization energy depending on the T_1–S_0 transition of (*S*)-BINAP. **(B)** The reorganization energy depending on the S_0–T_1 transition of (*S*)-H_8-BINAP. ((*S*)-BINAP = (*S*)-(−)-2,2′-bis(diphenylphosphino)-1,1′-binaphthyl; (*S*)-H_8-BINAP = (*S*)-bis(diphenylphosphino)-5,5′,6,6′,7,7′,8,8′-octahydro-1,1′-binaphthyl).

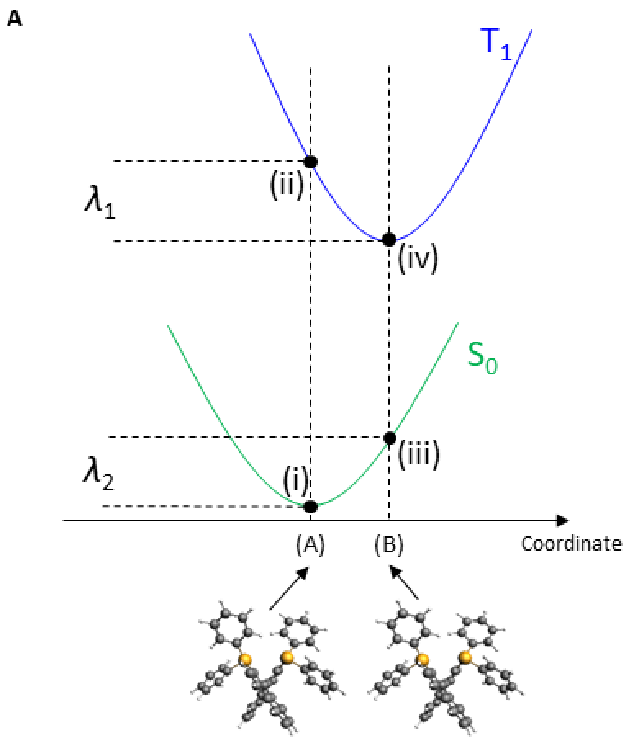

Supplementary Figure 12. Schematic procedure for calculating the reorganization energy of the energy transfer from the T_1 of (*S*)-BINAP to the T_1 of (*S*)-H_8-BINAP with the condition that (*S*)-BINAP and (*S*)-H_8-BINAP have no intermolecular interactions. **(A)** The reorganization energy depending on the T_1-S_0 transition of (*S*)-BINAP. **(B)** The reorganization energy depending on the S_0-T_1 transition of (*S*)-H_8-BINAP. ((*S*)-BINAP = (*S*)-(−)-2,2′-bis(diphenylphosphino)-1,1′-binaphthyl; (*S*)-H_8-BINAP = (*S*)-bis(diphenylphosphino)-5,5′,6,6′,7,7′,8,8′-octahydro-1,1′-binaphthyl).

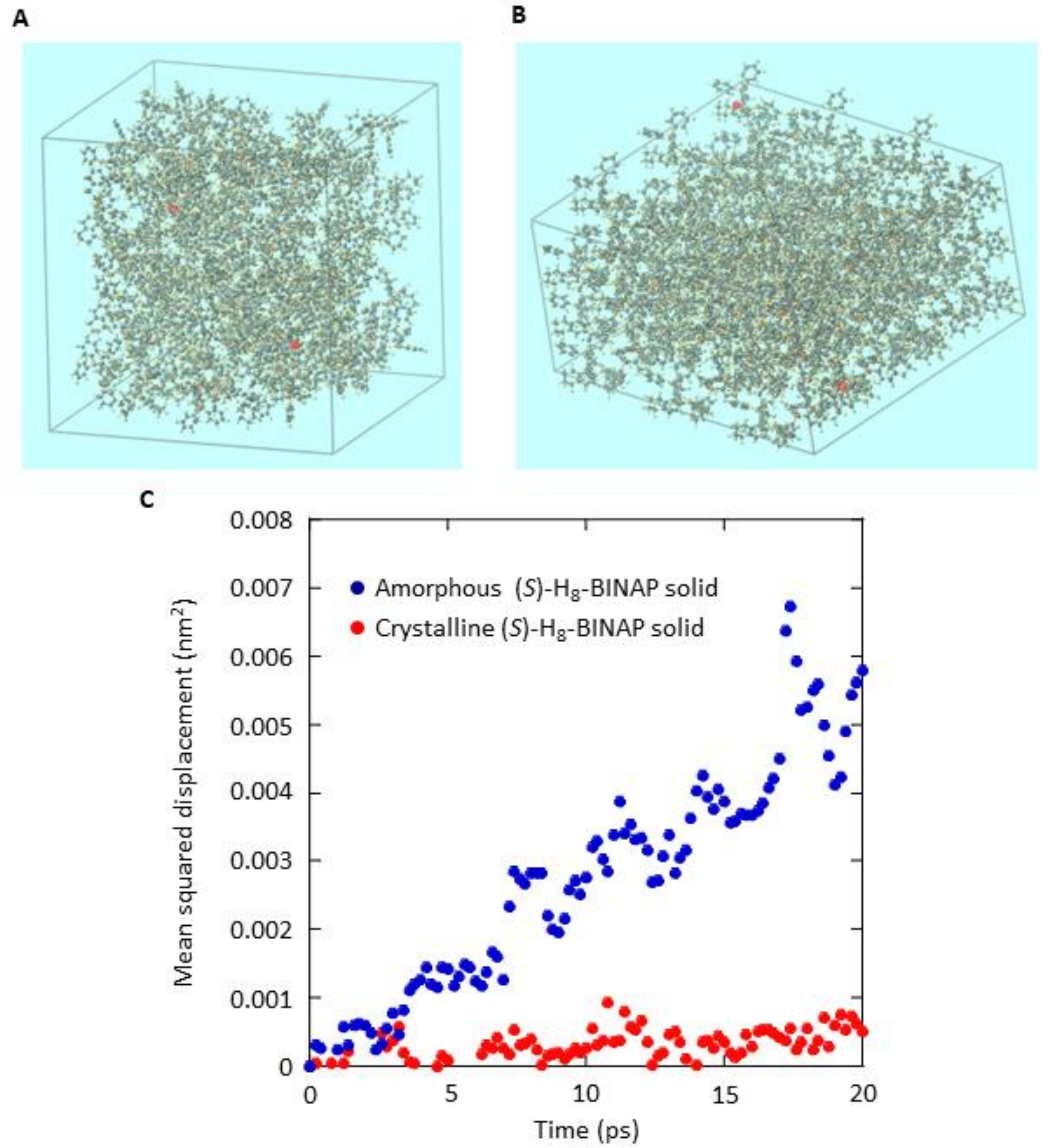

Supplementary Figure 13. Molecular dynamics simulation of the diffusion coefficient at RT of a solid comprising (*S*)-H_8-BINAP molecules. **(A)** Structure of 100 (*S*)-H_8-BINAP molecules in the amorphous state. **(B)** Structure of 108 (*S*)-H_8-BINAP molecules in the crystalline state. **(C)** Relationship between the mean square displacement at RT and time for an amorphous (*S*)-H_8-BINAP solid (blue) and a crystalline (*S*)-H_8-BINAP solid (red). The calculated *D*(RT) values were 2.7×10^{-7} cm^2/s for the amorphous (*S*)-H_8-BINAP solid and 2.7×10^{-8} cm^2/s for the crystalline (*S*)-H_8-BINAP solid. ((*S*)-H_8-BINAP = (*S*)-bis(diphenylphosphino)-5,5′,6,6′,7,7′,8,8′-octahydro-1,1′-binaphthyl; RT = room temperature).
